# When Fail-Secure Is Not Fail-Safe: Parser Safety in S-100 Maritime Navigation

**DOI:** 10.3390/s26206448

**Published:** 2026-10-12

**Authors:** Hoyeon Cho, Seojeong Lee, Changui Lee

**Affiliations:** 1 Division of Maritime Information Technology, National Korea Maritime and Ocean University, Busan 49112, Republic of Korea; hoyeoncho@g.kmou.ac.kr; 2 Division of Marine System Engineering, National Korea Maritime and Ocean University, Busan 49112, Republic of Korea; sjlee@kmou.ac.kr

**Keywords:** cyber–physical systems, ECDIS, HDF5, heap overflow, integer overflow, maritime cybersecurity, memory corruption, parser input validation, S-100 standard, S-102

## Abstract

Safety-critical maritime navigation depends on machine-readable data products. Their digital signatures establish provenance and integrity but do not guarantee that authenticated content can be parsed safely: even passive data containing no executable code can drive allocation through file-supplied metadata. Using OpenS100, an open S-100 reference implementation, this paper asks whether passive S-102 products can compromise parser memory safety and whether operating system protection alone preserves availability. This paper analyzed the S-102 bathymetric surface specification, built a structurally valid proof-of-concept file with inconsistent grid metadata, and ran it under strong and weak operating system protection across repeated trials. The file triggered an integer overflow and heap buffer overflow without an executable payload. Under strong memory protection, no tested exploit achieved code execution; process termination dominated and the base trigger produced deterministic denial of service, a Fail-Secure outcome that still removed the navigation display. Under weak protection, existing-function reuse achieved Arbitrary Code Execution (ACE) in 14 of 200 launches (7.0%). Neither preserved safe-navigation continuity in the tested application. A four-layer application-level validation sequence applied before allocation prevented both, with no workload-dominant overhead in three desktop microbenchmarks. Passive data are therefore security-relevant parser inputs; preserving both security and availability requires validation before corruption occurs.

## 1. Introduction

Maritime navigation is transitioning from the legacy S-57 standard to the International Hydrographic Organization (IHO) S-100 Universal Hydrographic Data Model, with S-100-based Electronic Chart Display and Information Systems (ECDISs) scheduled for voluntary adoption from January 2026 and mandatory installation in new equipment from January 2029. The transition is not a change of chart format; where S-57 described what a chart should display, S-100 describes a family of data products that together carry the safety information a bridge team acts on, including bathymetry in S-102, water levels in S-104, surface currents in S-111, and under-keel clearance management in S-129, with the family extending further into port and traffic management. That breadth was deliberate: S-100 was developed as the data framework on which the e-Navigation concept would be built. Its data products therefore provide information used directly in navigational decision-making rather than serving only as inputs to a display layer.

When ECDIS supplies depth information used in under-keel-clearance decisions, it functions as an Operational Technology (OT) system rather than merely as a presentation system. It is then a Cyber–Physical System (CPS) in the ordinary sense of computation coupled to a physical process, here through the navigator acting on what the system reports. Because ECDIS is the mandated primary means of navigation for certain vessel classes, losing the display during pilotage is itself a safety event, occurring while the vessel is still moving. This shift also changes what must be treated as security-sensitive. If the numbers a parser reads determine what the navigator sees and how much water the hull is believed to have under it, then those numbers warrant the same care as the code that draws them, and the safety of processing data becomes a question of the same order as the safety of executing a script.

Prior work established the code half of that question. The S-100 portrayal architecture interprets Lua scripts in portrayal catalogues as executable content, and malicious scripts enable Remote Code Execution (RCE) where sandboxing is absent, with the root cause traced to unconstrained script execution in the portrayal architecture [1]. The natural response is to harden script execution, and the assumption underlying that response is that passive content, which by definition contains no executable code, needs no equivalent hardening. This paper examines the data half, finds that this assumption does not hold, and turns the result into a validation pattern that can be applied before any allocation is made. Passive products are parsed rather than interpreted, but the parsing allocates memory from values the file itself supplies, which is enough to corrupt memory with no executable payload. A crafted S-102 file whose grid dimension metadata contradicts its own dataset triggers an integer overflow in the buffer size calculation, and a heap buffer overflow follows, while the file remains structurally valid at the HDF5 level. This paper did not sign and verify the proof-of-concept under S-100 Part 15; authenticated delivery is treated as a threat-model property, because content introduced before authorized signing or through compromised signing infrastructure would be signed validly without signature verification evaluating parser safety.

Parsing safety sits at a boundary that the S-100 architecture draws explicitly. Part 15 delimits data protection to provenance and transmission, addressing the protection of product information inside an end-user application at the Original Equipment Manufacturer (OEM) level [2], a clause quoted in Section 2.2. Drawing that boundary is a conventional choice for a data protection scheme. Accordingly, whether authenticated content can be parsed safely is addressed at the implementation level. That is where this paper looks. The immediate cause of the defect analyzed here is an unchecked integer multiplication in the OpenS100 parser, and the S-102 product specification mandates the metadata attributes that drive allocation without prescribing how those values should be checked before use. A parser that takes those values at face value can therefore reach the same state, and Section 3.2 sets out the delivery paths by which such a file reaches a vessel.

A similar assumption is often made about operating system protections. Just as a verified signature is often read as a warrant that content is safe to process, a hardened operating system is expected to contain whatever the parser gets wrong, so that platform-level memory protection absorbs the residual risk. The principal finding of this paper concerns what that layer actually delivers. The same vulnerability was tested under two operating system environments, a modern one with strong memory protection and a legacy one of the kind still encountered in deployed ECDISs. Under strong protection, the operating system (OS) detects the corruption and terminates the ECDIS process, blocking Arbitrary Code Execution (ACE) in a textbook Fail-Secure response from an Information Technology (IT) perspective; yet in an OT context, that same termination removes the primary navigation display while the vessel is still transiting hazardous waters. Under weak protection, once the exploitation strategy shifted from injected code to existing-function reuse, ACE was observed probabilistically, leaving the navigator operating a compromised system without indication. In each case, the OS did what it was designed to do, and in neither case did the maritime mission survive, because once memory corruption has occurred, OS-level intervention cannot simultaneously guarantee security containment and continued availability. The conclusion is therefore about placement rather than strength: validation has to occur before file-supplied values are used for allocation.

Four contributions follow. The paper demonstrates that passive S-102 products can corrupt memory in safety-critical navigation systems, extending the threat model of [1] beyond active content. It locates the enabling condition in a metadata-driven allocation pattern rather than in a single coding error, motivating a validation principle that may apply to other parsers using the same allocation structure; this broader applicability is a structural inference and was not experimentally validated for S-104, S-111, or commercial S-100 implementations. It characterizes the dual-outcome trap empirically across two protection regimes, showing that a defense placed after corruption cannot meet the maritime availability requirement, however reliably it detects the corruption. Furthermore, it proposes and verifies a four-layer application-level patch that removes both outcomes independently of OS-dependent mechanisms, at a measured overhead below per-repeat measurement noise.

Four bounds apply to these claims. The paper does not assert that any implementation violates the S-100 standard, since the specification does not prescribe the parser-side pre-allocation validation procedure absent from the analyzed implementation; it does not characterize any commercial ECDIS product, since none was tested; it does not treat the Part 15 scope boundary as a defect, since confining a data protection scheme to provenance and transmission is a conventional design choice; and its generalization to other S-100 product parsers is a structural inference from the shared grid structure, with empirical confirmation left to future work. What the paper offers is a validation pattern for metadata-driven allocation, together with the measured cost of applying it. Section 2 reviews background and related work, Section 3 presents the methodology and vulnerability analysis, Section 4 reports the experimental results, Section 5 proposes countermeasures, and Section 6 discusses limitations before Section 7 concludes.

## 2. Background and Related Work

### 2.1. Maritime Cyber–Physical Systems and Security Paradigms

Maritime navigation systems occupy a position at the intersection of IT and OT. ECDIS is a CPS in which software processing digital data directly influences physical navigation decisions, and a software failure carries into the physical world through the decisions the bridge team takes on its output. This dual nature creates tension between two design principles, which this paper uses according to their established definitions rather than as synonyms for security and availability. In NIST/CNSSI terminology, a Fail-Secure response is a mode of termination that prevents loss of a secure state when a failure is detected, even though that termination may itself deny service [3]. A Fail-Safe response, by contrast, is a transition into a state that prevents damage to protected resources or entities; in IEEE software-engineering terminology, a Fail-Safe system automatically enters a safe operating mode upon failure, whereas a system that retains partial operational capability after failure is more precisely termed Fail-Soft [4]. Availability and safety are distinct dependability attributes rather than the same property [5]. In enterprise IT, terminating a compromised process is an appropriate Fail-Secure response, since a crashed web server can be restarted and the cost is temporary unavailability. In an OT/CPS such as ECDIS, that same termination remains Fail-Secure at the process level, but whether the system as a whole reaches a Fail-Safe state is a separate, system-level question, because in industrial control systems, nuclear plants, and aviation, an unexpected shutdown can itself contribute to physical harm unless the surrounding system transitions to a safe state through backup, fault isolation, or graceful degradation.

The International Association of Classification Societies (IACS) Unified Requirements E26 on Cyber Resilience of Ships and E27 on Cyber Resilience of On-board Systems and Equipment, mandatory for new ships from July 2024, establish cyber resilience requirements that implicitly recognize this tension [6,7]. E26 sets incident-response and recovery provisions intended to preserve, as far as practicable, the functionality and level of service needed for safe ship operation during a cyber incident. That is an operational-continuity requirement, one contributor to a Fail-Safe navigation state. E27 mandates that equipment manufacturers demonstrate resilience against cyber threats, including the ability to detect anomalies and maintain essential functions. Both requirements focus on system-level resilience and anomaly detection, and neither treats input validation at the data-product parsing stage as a requirement item.

The International Maritime Organization (IMO) Safety of Life at Sea (SOLAS) Convention, Chapter V, Regulation 19, mandates ECDIS as the primary means of navigation for certain vessel classes [8]. When ECDIS is the primary navigation system, its availability is a legal requirement for safe navigation rather than a convenience. IMO ECDIS performance standards require independent backup arrangements so that safe navigation is not compromised in the event of ECDIS failure, including timely transfer to the backup system during critical navigation situations [9]. The requirement for timely transfer during critical navigation situations indicates that continuity and transition time are themselves safety-relevant properties of ECDIS failure handling, since the navigator must recognize the failure, transfer to backup, and re-establish situational awareness while the vessel continues to move.

The conflict admits two symmetric forms. First, what IT security considers successful defense, namely process termination to prevent code execution, is what maritime OT considers a serious failure, namely loss of the primary navigation system. An attacker who understands this conflict need not achieve ACE at all. It is sufficient to trigger a condition that causes the IT-oriented OS to terminate the navigation process, and the OS performs the Denial of Service (DoS) on the attacker’s behalf. Second, when OS protection is absent or weakened, as in legacy deployments still common in the maritime fleet, the same vulnerability reaches ACE through probabilistic exploitation, producing a different but equally severe form of safety violation in which the navigator continues to operate a compromised system without indication. The two forms appear at opposite ends of OS protection strength but share a common structural feature. Both occur because the defense operates after memory corruption has already occurred, at which point any OS response, whether termination or non-detection, has already failed to protect the maritime mission. This dual-outcome structure is empirically demonstrated in Section 4 and motivates the application-level mitigations proposed in Section 5.

### 2.2. The S-100 Ecosystem and S-102 Bathymetric Surface

The IHO S-100 standard provides the technical foundation for next-generation ECDISs. S-100 was developed to overcome the rigid data structure and maintenance limitations of the legacy S-57 standard and aligns with the International Organization for Standardization (ISO) 19100 series of geographic information standards. The standard extends beyond defining encoding formats by providing a flexible framework for developing various marine-related data product specifications.

As Section 1 noted, the S-100 family extends well beyond chart display, and its gridded products supply values that navigation decisions are taken from directly. That breadth is what makes parsing safety, rather than script execution alone, a security question for this ecosystem.

S-101 Electronic Navigational Chart (ENC) replaces the legacy S-57 format with additional chart features for primary navigation, while S-102 Bathymetric Surface, S-104 Water Level Information, S-111 Surface Currents, and S-129 Under Keel Clearance Management address information that S-57 did not cover. Each product specification defines its own data model, encoding format, and metadata requirements while sharing common S-100 framework elements. S-102 Bathymetric Surface, the case examined in this paper, provides high-resolution gridded bathymetric data for precise depth information beyond what traditional ENCs offer. The product specification uses Hierarchical Data Format version 5 (HDF5) as its encoding format, storing depth measurements and associated uncertainty values in a regular grid structure. An S-102 file contains metadata attributes including grid dimensions specified by *numPointsLatitudinal* and *numPointsLongitudinal*, defining the number of depth points along each axis. The parser reads these dimension values from file metadata and uses them to allocate memory for storing the bathymetric grid data.

**Figure 1:**
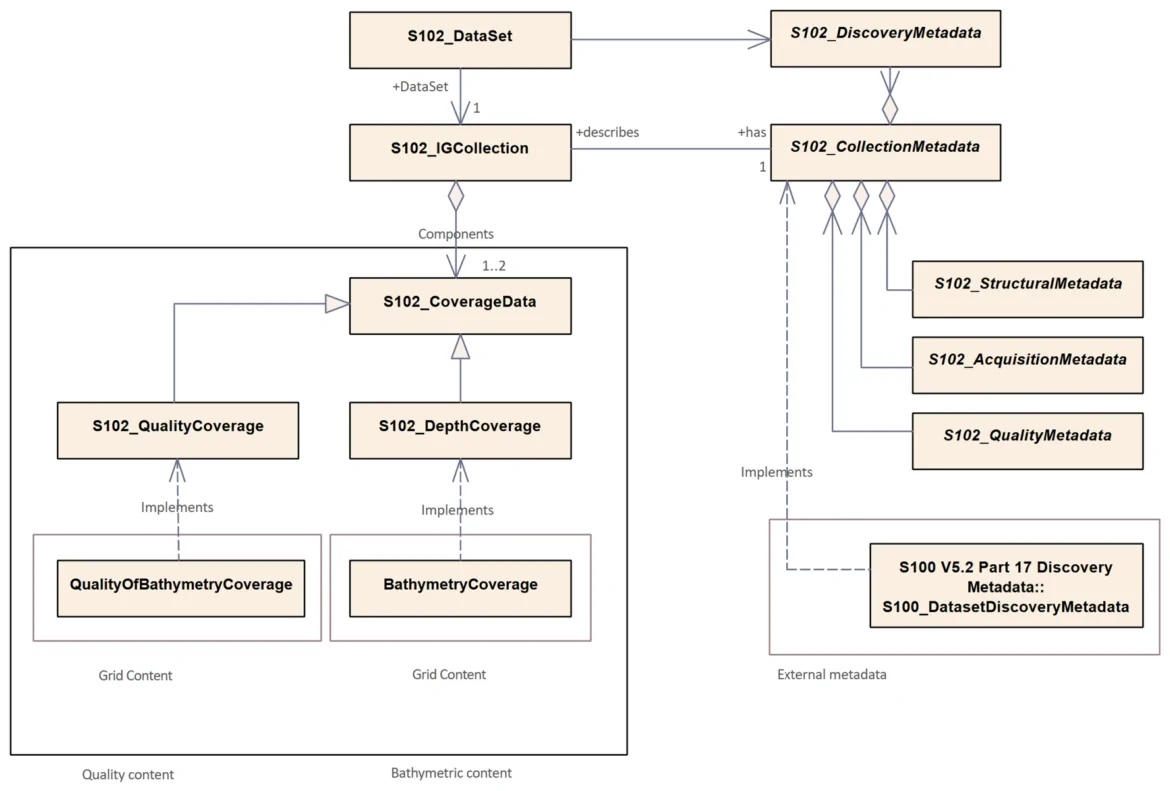
S-102 logical data model and HDF5 layout.

Figure 1 illustrates the S-102 logical data model and hierarchical structure. The S102_DataSet contains S102_IGCollection, which aggregates S102_CoverageData instances. Each BathymetryCoverage group contains one or more coverage instances with grid dimension attributes and a values dataset storing depth and uncertainty measurements. During parsing, the parser reads dimension attributes from this structure, calculates the required buffer size by multiplying dimensions, allocates memory, and reads depth values into the allocated buffer. This creates a dependency where the memory allocation size is determined by metadata values within the file being parsed, a property exploited by the vulnerability analyzed in Section 3.

The S-100 security architecture addresses data authenticity through Part 15, the Data Protection Scheme, and the International Electrotechnical Commission (IEC) 63173-2 Secure Communication Between Ship and Shore (SECOM) standard [2,10]. Part 15 specifies procedures for generating and verifying digital signatures that ensure data products originate from authorized sources and remain unmodified during transmission. However, Part 15 explicitly limits its own scope. It states, “The security scheme does not address how the product information is protected once it is within an end-user application. This is the responsibility of the Original Equipment Manufacturers (OEMs)” [2]. The standard authenticates the data and specifies the data model, and how safely that content is then parsed is addressed at the OEM implementation level.

S-102 defines the required relationship between the dimension metadata and the *values* dataset, but neither it nor Part 15 prescribes the parser-side validation procedure, such as bounds checking, safe integer arithmetic, or a pre-allocation metadata-to-dataspace consistency check, by which an implementation must verify that relationship before allocation or *H5Dread*. The authentication layer and the parsing-safety layer therefore address complementary concerns, and signature verification carries no implication about parser behavior. The implementation analyzed in Section 3 illustrates why parser-level validation remains relevant at this interface.

### 2.3. Memory Corruption, Mitigations, and Exploitation Techniques

#### 2.3.1. Integer Overflow and Heap Corruption

Integer overflow occurs when arithmetic operations produce results exceeding the maximum value representable in the destination data type. For signed 32-bit integers, values exceeding 2,147,483,647 constitute undefined behavior in C/C++ [11], meaning the compiler may produce arbitrary results. When the result of a size calculation overflows, the program may allocate a buffer much smaller than intended. Subsequent operations expecting the larger size then write beyond the undersized buffer, causing heap buffer overflow, a condition where data overwrites adjacent heap metadata and potentially corrupts program control structures such as function pointers and object pointers. The vulnerability documented in this paper follows this classical pattern, namely Common Weakness Enumeration (CWE) 190 leading to CWE-122, but specifically through metadata-controlled allocation in a parser context, where the overflowing factors originate from authenticated input data.

#### 2.3.2. OS-Level Mitigations

Modern operating systems implement multiple protection mechanisms against memory corruption exploitation. Address Space Layout Randomization (ASLR) randomizes the memory locations of code and data regions, making it difficult for attackers to predict addresses needed for exploitation. ASLR entropy varies by OS generation. Legacy Windows configurations provide substantially lower heap-base entropy than Windows 10 and later, so a single planted spray base matches the live heap far more often on a legacy system than on a modern one. Data Execution Prevention (DEP) marks data memory regions as non-executable, preventing direct shellcode execution. Heap protection mechanisms including Low Fragmentation Heap (LFH) randomization, heap walker integrity checking, and termination-on-corruption provide additional barriers, the last of these being a Windows kernel heap policy that can be active for a process. When heap integrity checks detect corruption, the operating system terminates the process to prevent further exploitation. This termination behavior, designed to block ACE, becomes central to the dual-outcome trap examined in Section 4.

These protections operate at different levels with different controllability. ASLR and DEP are enabled through compiler and linker flags that application developers can configure. In contrast, heap integrity checking is implemented within the operating system kernel, and applications cannot disable these kernel-level protections regardless of how they are compiled. This distinction becomes important when analyzing the IT/OT security conflict, because the protection mechanism that blocks code execution and causes process termination is precisely the mechanism that developers cannot control or customize for safety-critical operational contexts.

A separate property, often unstated in vulnerability analyses but critical to this paper, is the architectural separation between heap and stack memory regions. On x86-64 platforms, the heap and the stack occupy distinct virtual memory ranges, and a heap buffer overflow can corrupt heap-resident objects but cannot influence stack contents. When indirect-call sites load function arguments from stack-derived registers, for example, via *mov rcx*, *rbx* where RBX holds a stack pointer, heap-overflow primitives cannot corrupt the argument value. Heap–stack isolation is not a configurable mitigation but an architectural property of the memory model itself, and it is one of the three independent defenses analyzed in Section 4.3.

#### 2.3.3. Exploitation Techniques for Indirect-Call Hijacking

When a heap-overflow primitive corrupts an object pointer that is later dereferenced, the attacker can redirect the indirect call to attacker-chosen code. Three families of techniques have evolved to bridge the gap between memory corruption and reliable code execution.

NOP sleds, introduced in early stack-smashing exploits and generalized by [12], increase the probability that an injected jump lands within attacker-controlled code by filling the spray region with No-Operation (NOP) instructions, typically 0x90. When the corrupted instruction pointer lands anywhere within the sled, execution slides forward into the shellcode payload. NOP sleds remain effective when the injected value is an instruction pointer targeting executable memory, but they fail in modern environments for two reasons. First, DEP prevents execution of data regions, eliminating the destination shellcode. Second, when the corruption manifests as a data pointer that the program subsequently dereferences, rather than as an instruction pointer that the CPU directly executes, NOP fill produces invalid pointer values, and dereferencing 0x9090909090909090 traps as an access violation before any sled-like behavior can occur. Multi-level dereference chains, where the program follows two or more pointer hops before the indirect call, amplify this failure, because every hop must yield a valid pointer or the chain terminates in a mid-chain access violation.

Heap spray techniques [13,14] address the predictability of pointer targets rather than instruction targets. By filling large heap regions with attacker-controlled patterns and choosing a target address with high prevalence, such as 0x0c0c0c0c on 32-bit systems or 0x10000008 on 64-bit, the attacker increases the probability that a corrupted pointer happens to point into the spray region. Heap spray is content-agnostic, so the spray bytes can be NOP sleds for direct execution, shellcode, Return-Oriented Programming (ROP) gadget addresses, or other structured payloads. However, when the corruption triggers a multi-level dereference chain, heap spray alone is insufficient, because it is not enough for the initial pointer to land in the spray; every level of the chain must find a valid pointer at the dereferenced offset.

Pointer fill addresses the multi-level dereference problem directly. Rather than filling spray slots with NOP bytes, the technique fills every 8-byte slot of the spray block with a pointer back into the spray region itself, so that every dereference along the chain reads a valid address regardless of where in the block it lands. The construction used in this paper, which this paper calls *Pointer Sled*, applies this idea to the chain reached through HDF5-driven heap corruption in OpenS100. Its spray block repeats a fixed cycle at every 32-byte alignment, and each cycle carries a command string, a spray-base pointer, an argument pointer, and the calibrated address of the target library function, so that the chain finds a usable value at whichever alignment the allocator happens to place it. Concrete offsets and addresses, together with the full construction and its per-variant parameters, are withheld under coordinated disclosure. This paper does not claim it as a wholly new exploitation class, since it adapts existing pointer-fill, fake-object, and pointer-spray ideas. Its relevance here is that NOP-style byte filling fails before instruction dispatch because the corrupted value is dereferenced as data, whereas pointer filling preserves mid-chain validity, which is what makes the weak-protection outcome of Section 4.4 reachable at all.

These techniques are subject to the OS-level mitigations described in Section 2.3.2. ASLR lowers the per-trial probability that a single planted spray base matches the actual heap base, from near-certainty on legacy Windows to a very small probability on Windows 10 and later. Termination-on-corruption raises an additional barrier by detecting widespread spray patterns before the chain can complete, creating a trade-off between matching probability and detection probability. Heap–stack isolation provides a final architectural barrier when indirect-call sites load arguments from stack-derived registers. The interaction of these three defenses with pointer-fill spraying is the empirical subject of Section 4.

#### 2.3.4. Documented Parser Vulnerabilities and Automated Defenses

Memory corruption in file parsers is a well-documented class. Image format parsers for Portable Network Graphics (PNG), Joint Photographic Experts Group (JPEG), and Tagged Image File Format (TIFF) have yielded Common Vulnerabilities and Exposures (CVE) entries for integer overflow in dimension handling, and CWE-190 Integer Overflow or Wraparound documents the pattern in which arithmetic produces results exceeding representable bounds, with critical severity when those results control allocation sizes [15]. The SEI CERT C/C++ Coding Standard addresses the same pattern through rules such as INT32-C [16], and MISRA C:2012 [17] prohibits constructs prone to undefined behavior, including unchecked integer arithmetic. Maritime software development has not systematically adopted either, and no maritime-specific secure coding standard addresses parser security requirements. The HDF5 library used by S-102 has its own history, since CVE-2017-17505 through CVE-2017-17509 documented heap buffer overflow and NULL pointer dereference vulnerabilities in version 1.10.1, and CVE-2021-37501 identified an integer overflow in *H5O__attr_decode()* where ds_size * dt_size truncated to zero and produced a severely undersized allocation, a pattern directly analogous to the one analyzed in this paper.

Automated tooling exists for both detection and repair. INTGUARD [18] employs static symbolic execution and SMT solving to locate integer overflow sites in source code and generate multi-precision repairs, while IntPatch [19] uses dataflow analysis on the LLVM intermediate representation to instrument programs with runtime overflow checks that prevent integer-overflow-to-buffer-overflow chains. Either approach would have prevented the vulnerability documented in this paper. Coverage-guided greybox fuzzers such as AFLNet [20] have likewise been effective at discovering parser vulnerabilities in complex file formats, and adapting them to mutate HDF5 metadata fields could systematically uncover boundary conditions that manual review may miss. No published work has applied such techniques to maritime data parsers, and this distance between available tooling and maritime software practice is itself part of the problem this paper describes.

### 2.4. Related Work

Maritime cybersecurity research has grown substantially following the IMO MSC.428(98) resolution mandating cyber risk management [21]. Li et al. conducted a comprehensive review of maritime cyber attacks, presenting a taxonomy covering ECDIS, Automatic Identification System (AIS), Global Navigation Satellite System (GNSS), Voyage Data Recorder (VDR), and Global Maritime Distress and Safety System (GMDSS) vulnerabilities, identifying third-party components and outdated operating systems as primary ECDIS security concerns [22]. Svilicic scanned the navigational systems of three vessels in international service and found Windows 7 and Windows Embedded Standard 7 still running an integrated navigation system and an ARPA, with the obsolete SMBv1 service enabled on both [23]. A separate scan of ECDIS installations by the same group traced the risk to the underlying operating system and to third-party applications [24]. Jo et al. analyzed ship equipment cyberattack scenarios using the MITRE ATT&CK framework, finding Server Message Block (SMB) and Remote Desktop Protocol (RDP) vulnerabilities across multiple manufacturers’ ECDIS platforms [25]. These field surveys document that legacy operating systems, including Windows 7 and earlier, remain in operational use across the maritime fleet, which justifies the dual-environment experimental design adopted in this paper, since a range of OS generations coexists in the deployed ECDIS population and both modern and legacy protection levels are present at the same time. These studies document platform-level vulnerabilities but do not examine whether navigation data products themselves can serve as attack vectors.

Prior work by the authors demonstrated that the S-100 portrayal engine contains vulnerabilities enabling RCE through malicious Lua scripts in portrayal catalogues [1]. That research established that executable content within S-100 data products poses security risks when script execution environments lack proper sandboxing. That finding subsequently entered the IHO technical process: S-100TSM12 noted this concern—recorded as raised by the present authors at KMOU and demonstrated by OSI—highlighted that OEMs should mandate execution checks of Lua scripts, and recommended that the S-100WG establish a review process for Lua maintenance [26]. A subsequent S-100WG11 proposal from the United States cited that work in recommending migration of the S-100 Part 13 Lua baseline from Lua 5.1 to Lua 5.5 and the establishment of a formal process for evaluating scripting-language versions [27]. This paper continues that line of research, moving from identifying where risk arises to constructing and evaluating a defense: it examines whether passive data products, containing no executable scripts, can affect navigation systems through parsing, and what validation prevents it.

The risk is amplified by the structure of maritime data distribution. Navigation data products traverse a multi-tier distribution infrastructure before reaching vessel systems. Hydrographic Offices produce authoritative data products, signing each product with IHO-issued certificates. Regional ENC Coordinating Centres (RENCs) such as PRIMAR and IC-ENC aggregate and redistribute products, the IHO Infrastructure Centre centralizes distribution of catalogues, and commercial Value Added Resellers (VARs) further distribute products to end users. A pure downstream-tampering attack at the redistribution tiers is detected at signature verification, so it is not a viable delivery path on its own. The realistic injection points are pre-signing pipeline compromise at a hydrographic office, signing-infrastructure or key compromise that lets the attacker sign their own malicious payload, and operational paths that load files without full signature validation. The S-100 Part 15 authentication mechanism verifies source authenticity but not content safety [2]. A legitimately signed data product from an authorized producer can still contain dimension values that trigger parser vulnerabilities, because the signing infrastructure has no mechanism to evaluate parsing safety. The detailed scenario decomposition is presented in Section 3.2.

The literature reveals a gap at the intersection of three domains, namely memory corruption vulnerabilities in data parsers, maritime navigation data products, and the IT/OT security paradigm conflict. No published work has investigated whether passive S-100 data products can trigger memory corruption in parsing implementations, how such behavior relates to the allocation pattern the product specifications describe, or how OS-level exploit mitigations designed for IT environments affect the safety and availability of maritime CPS across the spectrum of OS protection strength present in the deployed fleet. This paper addresses all three aspects.

## 3. Methodology and Vulnerability Analysis

### 3.1. Analysis Methodology

Establishing whether a passive data product can compromise a parser requires more than exhibiting a crash. It requires showing that the crash yields an exploit primitive rather than a defect-class fault, that the consequence depends on the deployment environment, that the layer positioned to prevent it can be identified, and that the defect can be closed there. The analysis in this paper is organized as five stages that answer those questions in sequence.

The first stage is a specification review. This paper reads the S-102 Product Specification Edition 2.2.0 and S-100 Part 15 against each other to locate the point at which memory allocation is driven by file-supplied metadata and to determine what validation, if any, either document prescribes at that point. The second stage constructs a threat model that places the resulting exposure in the S-100 distribution infrastructure, identifying which delivery paths can carry a malicious product to a vessel and which security gate each path must pass. The third stage builds a Proof-of-Concept (PoC) file that is structurally valid, passes HDF5 conformance tooling, and carries metadata that contradicts its own dataset, so that the trigger depends on the unvalidated metadata itself rather than on malformed encoding.

The fourth stage is a dual-environment experiment. Because the consequence of memory corruption is determined by the operating system rather than by the parser, the same PoC is executed under a modern protection stack and under a legacy one, and the outcome of each is measured across repeated trials rather than reported from a single run. This design is what allows the paper to distinguish a claim about the vulnerability from a claim about any particular platform. The fifth stage develops and verifies an application-level patch against the experimentally exercised overflow path and against benign products for functional regression, across the two environments to confirm that its effect does not depend on OS behavior, while the metadata–dataspace mismatch path is evaluated through source-level analysis.

The remainder of this section carries out the first three stages. Section 4 reports the fourth, and Section 5 the fifth.

### 3.2. Threat Model

The threat model considers an adversary capable of distributing malicious S-102 data products through legitimate channels. This threat aligns with the supply-chain attack patterns discussed in Section 1, where adversaries compromise authoritative distribution infrastructure to inject malicious content into trusted channels.

The adversary’s objective is to compromise ECDIS installations by crafting S-102 files that trigger memory corruption during parsing. The attacker possesses knowledge of the S-102 file format, the HDF5 encoding structure, and the target parser implementation. The attacker can create files with arbitrary metadata values, including grid dimensions that cause integer overflow when multiplied, and arrange for these files to be distributed through channels that vessels use to obtain bathymetric data. Notably, full compromise of a hydrographic office signing key is not the only viable path. Depending on the scenario, a crafted file may be introduced before signing through production-system compromise, signed through compromised signing infrastructure, or loaded through local workflows where signature verification is not fully enforced.

Two attack outcomes are considered. ACE allows the attacker to execute arbitrary commands on the target system. DoS crashes the navigation system, potentially during critical navigation phases. As Section 4 demonstrates, both outcomes occur with different rates depending on operating system protection strength, where DoS is deterministic under strong protection while ACE becomes probabilistically achievable under weak protection, but from a maritime CPS perspective, both outcomes are inconsistent with the availability and continuity properties required for a Fail-Safe navigation state.

The prior knowledge required of the attacker differs sharply between the two outcomes. Triggering the parser corruption requires knowledge of the S-102 encoding and of the vulnerable metadata-driven allocation path, but it does not require any knowledge of runtime memory addresses; crafted dimension metadata alone is sufficient, which is why the DoS outcome is deterministic. Escalating the same corruption to ACE in the tested Windows 7 environment requires additional target-specific information, since the pointer-fill strategy of Section 2.3.3 depends on a calibrated address for the reused library function and on a spray configuration compatible with the heap layout of the current environment. ASLR therefore bears on the ACE path rather than on the existence of the vulnerability. Because the calibrated address and a usable heap alignment are environment- and session-dependent, the exploit used here was recalibrated for the tested system after reboot, and this paper does not assume that a single universal address can be reused across arbitrary ECDIS installations. The ACE path additionally presupposes that the attacker knows the specific parser implementation and build in use, since the calibrated call-target address and the compatible spray layout are tied to that binary and its runtime environment; an attacker without this implementation- and environment-specific knowledge cannot be assumed to reproduce the measured weak-protection ACE rate. The demonstrated ACE result should accordingly be read as evidence that the memory-corruption primitive can be escalated within a sufficiently characterized weak-protection environment, not as a portable one-file exploit against unknown targets.

Table 1 illustrates the end-to-end attack chain from supply-chain compromise to navigation system failure, showing how a crafted S-102 file traverses the authenticated distribution infrastructure and exploits the parser vulnerability.

A signed-malicious-file delivery requires one of three viable scenarios, because downstream redistribution alone cannot modify a signed payload without invalidating the cryptographic signature. Adversaries with supply-chain compromise capability [28,29] can reach all three. The first is pre-signing pipeline compromise at a hydrographic office, in which the malicious payload is incorporated before signing and is therefore signed legitimately by the authorized producer. The second is signing infrastructure or key compromise, in which the attacker obtains the ability to issue signatures over their own malicious payload. The third comprises operational paths that load files without full signature validation, such as Universal Serial Bus (USB) import workflows where signature verification is configured for local trust or is not enforced for a given input path. Stage 3 of Table 1, marked “Signature valid”, applies to the first and second scenarios. The third bypasses Stage 4 entirely, because the verification gate is not enforced for the relevant input path. A pure downstream-tampering attack that modifies an already-signed product is detected at Stage 4 and is therefore not a viable delivery path on its own.

**Table 1:** End-to-end attack chain with ATT&CK for ICS mapping. Stage 4 denotes a threat-model condition under the pre-signing or compromised-signing scenario, not an experimentally reproduced signing-and-verification result.

Stage	Action and Security Gate	ATT&CK for ICS [30]
1	Craft malicious S-102 file, no gate	—
2	Inject file into supply chain, gated by SECOM signing	T0862 Supply Chain Compromise
3	RENC/VAR redistributes product, signature valid in the signing-pipeline scenario	—
4	Signature verification passes (threat-model condition, not reproduced)	—
5	Parser computes size with no overflow check	T0863 User Execution
6	Undersized buffer and heap overflow, no bounds check	—
7	OS response, terminating under strong protection and undetected under weak protection	T0814 Denial of Service
8	Navigation display loss or compromise, a maritime safety violation	T0829 Loss of View, T0827 Loss of Control

These three scenarios require different levels of attacker access and are not equally easy to realize. Pre-signing pipeline compromise requires access to an authorized data-production workflow at a hydrographic office, so that the payload is incorporated before legitimate signing. Signing-infrastructure or key compromise requires control over trusted signing credentials or the signing service itself, a stronger prerequisite than reaching a production workflow. Local import without enforced signature verification requires a deployment in which a given input path is treated as locally trusted, a condition that is implementation- and configuration-dependent and cannot be assumed to exist in every ECDIS product. This paper did not measure the prevalence or likelihood of these conditions, and no operational S-100 distribution infrastructure was accessed or compromised. The scenarios therefore bound where the demonstrated parser defect could matter; they do not establish that unrestricted injection of malicious data into a deployed ECDIS environment is generally feasible.

Compromise of an upstream trusted distribution path is a demanding attacker capability, but it is not without real-world precedent. In the 2016 compromise of the Republic of Korea military network, malicious code propagated into military intranet systems through compromised trusted internal infrastructure, illustrating that even a high-assurance environment can be reached when upstream infrastructure is breached [31]. In the SolarWinds incident, malicious code was inserted into the Orion software lifecycle before release, so that the trojanized components were digitally signed by the vendor and distributed through the normal update channel [28,32]. Operation ShadowHammer demonstrated a closely related case in which trojanized ASUS Live Update packages carried legitimate vendor signatures and were distributed from official update servers [29]. These incidents do not establish that S-100 signing or distribution infrastructure has been compromised, nor do they imply that arbitrary downstream modification can bypass Part 15 verification. Rather, they demonstrate the narrower threat-model premise relevant here: compromise upstream of, or within, a trusted production and distribution boundary can allow malicious content to reach end systems through channels that would ordinarily be treated as trustworthy.

The ATT&CK for ICS mapping reveals three observations about the attack chain. First, Stage 5 maps to T0863 User Execution because the act of opening a file, whether manual or through ECDIS auto-loading, triggers the parser that executes the attack. This contrasts with the Lua scripting vulnerability in prior work [1], which maps to T0853 Scripting, illustrating how the distinction between active content, namely script execution, and passive content, namely user execution, is reflected at the ATT&CK technique level. Second, the tactic separation between Stage 7 and Stage 8 captures the IT/OT paradox within the framework itself. T0814 Denial of Service belongs to the Inhibit Response Function tactic, representing the suppression of the system’s ability to respond, while T0829 Loss of View and T0827 Loss of Control belong to the Impact tactic, representing the operational consequence. The fact that the same OS response event spans both tactics confirms the location problem introduced in Section 1, since a single event carries simultaneous meaning at the defense-function level and the operational-impact level. Third, under ATT&CK Enterprise, the same attack would be classified as T1499 Endpoint DoS, an availability concern, whereas ATT&CK for ICS classifies the outcome as T0829 Loss of View, signifying that the operator has lost visibility into the physical process. This difference in classification demonstrates that the choice of threat framework itself determines how the severity of an attack is perceived.

### 3.3. Where Validation Is Left to the Implementation

Scoping application-internal protection outside a data protection scheme is a conventional and defensible boundary, and comparable security schemes make the same choice. The point of the review below is not that Part 15 draws this boundary, but that the validation which keeps metadata-driven allocation safe currently sits on the implementation side of that boundary. The analysis therefore describes the interface between specification and implementation rather than faulting any single document.

This paper conducted a systematic review of the S-102 Product Specification Edition 2.2.0, focusing on Section 10.2 on the BathymetryCoverage feature type, Section 10.2.1 on attribute definitions, and Section 12.3 on data quality requirements. The review identified the point at which file-supplied values are used to determine memory allocation. Section 10.2.1 defines *numPointsLatitudinal* and *numPointsLongitudinal* as mandatory integer attributes specifying grid dimensions. The specification mandates their presence and data types. Four aspects of their use are left unspecified.

- **Metadata-to-data consistency.** Verifying these metadata values against the actual HDF5 dataset dimensions, obtainable via *H5Sget_simple_extent_dims*, is an implementation-level operation.
- **Safe integer arithmetic.** How dimension values are turned into buffer sizes is not specified, and size calculations of this shape carry the well-documented overflow risk classified under CWE-190.
- **Bounds on dimension values.** No upper bound accompanies the attributes, while Section 11.2.2 recommends approximately 600×600 grid cells for practical file sizes, so the working range is indicated rather than constrained.
- **Parser-side validation in Part 15.** Consistent with its stated scope, Part 15 does not prescribe implementation-level input validation, bounds checking, or buffer-size verification.

Two consequences follow. The specification defines the data relationship but does not prescribe a parser-side pre-allocation validation procedure for enforcing it. Furthermore, the immediate cause analyzed here, an unchecked integer multiplication, is one instance of a broader failure mode that can arise in any parser using the same metadata-driven allocation pattern. That is what makes the validation sequence developed in Section 5.1 worth stating as a general pattern rather than as a fix to one function.

This interpretation is consistent with the scope Part 15 states for itself, quoted in Section 2.2, namely that the security scheme does not address how the product information is protected once it is within an end-user application. Part 15 and the S-102 product specification address complementary concerns, and the validation examined here belongs to the implementation layer that sits between them. Should current revision cycles already be considering implementation guidance in this area, the present analysis may serve as empirical input to it.

### 3.4. From Integer Overflow to Heap Corruption

OpenS100, developed based on the S-100 viewer of the Korea Hydrographic and Oceanographic Agency (KHOA) under the Ministry of Oceans and Fisheries, is an openly available reference implementation for S-100 data products [33]. It is published as open source and has been forked, so it is read and reused by other implementers, which is why its parsing code is a useful subject for this kind of analysis. The current analysis focuses on OpenS100 commit 2b1dafc1 dated 29 April 2026. The vulnerable function is designed to read gridded bathymetric depth and uncertainty data from S-102 product files, where each grid cell contains a depth value and an associated uncertainty value stored in HDF5 compound data types. The grid dimensions determine how much data must be read from the file into memory.

The vulnerability is triggered when an ECDIS application opens an S-102 file. The parser navigates to the BathymetryCoverage group, retrieves grid dimensions via *getNumPointsLatitudinal()* and *getNumPointsLongitudinal()*, and passes them to the vulnerable grid reader function. Inside this function, the expression *numLat* × *numLon* × *2* computes the buffer size using 32-bit arithmetic. When this calculation overflows, it produces a small value, and the subsequent *new float[bufSize]* allocates an undersized buffer. Finally, *H5Dread()* reads the full dataset into this undersized buffer, causing heap buffer overflow. This vulnerability constitutes a CWE-190 Integer Overflow leading to CWE-122 Heap-based Buffer Overflow chain. Table 2 summarizes the conditions required to trigger the vulnerability.

Listing 1 is reproduced to support the analysis above. The code reads the mandated attributes, uses them for allocation, and calls HDF5 as the specification describes. The validation steps that would prevent the outcome analyzed below are not among the things it prescribes.

**Listing 1.** Vulnerable S-102 grid reader function in OpenS100 (commit 2b1dafc1).// S102_VG_BathymetryCoverage.cpp:20–48 bool S102_VG_BathymetryCoverage::Read(     hid_t groupID, int numLat, int numLon) {     auto dsID = H5Dopen(groupID, "values", H5P_DEFAULT);     if (dsID < 0) {return false;}       auto type = H5Dget_type(dsID);     if (H5Tget_class(type) == H5T_COMPOUND) { // [0] guards all         // Unchecked 32-bit multiplication (analyzed below)         auto bufSize = numLat ∗ numLon ∗ 2;     // [1] wraps to 32         auto size = numLat ∗ numLon;              // [2] wraps to 16           float ∗ buf = new float[bufSize];          // [3] 128 bytes         depth = new float[size];         uncertainty = new float[size];         memset(buf, 0, bufSize ∗ sizeof(float));         memset(depth, 0, size ∗ sizeof(float));         memset(uncertainty, 0, size ∗ sizeof(float));           H5Dread(dsID, type, H5S_ALL, H5S_ALL, // [4] Overflow                     H5P_DEFAULT, buf);           for (int i = 0; i < size; i++) {               depth[i] = buf[i ∗ 2];               uncertainty[i] = buf[(i ∗ 2) + 1];         }         delete[] buf;     }     return true;                                       // [5] unconditional }

At line [1], the C++ *auto* keyword infers type *int* from the integer operands, and the multiplication is performed in 32-bit signed arithmetic. When the mathematical result exceeds *INT_MAX*, that is 2,147,483,647, signed overflow occurs, which constitutes undefined behavior in C++ [34]. The wraparound values reported here are what the tested MSVC 19.42 build empirically produced and are not a language-guaranteed result. With numLat=4 and $\mathrm{numLon}=2^{30}+4$, the inner product at line [2], numLat×numLon, wraps to 16 in 32-bit signed arithmetic and the buffer-size value bufSize=numLat×numLon×2 wraps to 32. At line [3], *new float[bufSize]* therefore allocates only 32×sizeof(float)=128 bytes. At line [4], *H5Dread* reads the full 8192-byte, or 8 KB, dataset regardless of buffer size, overflowing the 128-byte allocation by 8192−128=8064 bytes and corrupting adjacent heap memory.

The underlying vulnerability is the absence of any coupling between the buffer size computed from metadata and the actual dataset size that *H5Dread* writes. The *H5S_ALL* parameter instructs the HDF5 library to read the entire dataset as defined by the file’s own dataspace structure, independently of any application-level size calculation. This decoupling produces two distinct attack paths that both result in heap buffer overflow. In the primary path, namely Case 1, used by the PoC in this paper, the attacker selects dimension values whose product overflows the 32-bit integer range, causing the parser to allocate a buffer far smaller than intended. A secondary path, namely Case 2, is also possible, where the attacker declares genuinely small dimensions such as a 2×2 grid but embeds a dataset whose actual size far exceeds this allocation, exploiting the metadata–dataspace mismatch without requiring arithmetic overflow. The four-layer patch proposed in Section 5.1 addresses both cases. Layers 1–3 defend against Case 1 through input validation, checked arithmetic, and size limits, while Layer 4, the HDF5 cross-validation layer, defends against Case 2 by verifying that declared metadata matches the actual dataspace dimensions before any read operation. All four layers are part of the recommended patch.

A third path involves neither the size calculation nor the dataspace. The compound-type test at line [0] of Listing 1 guards the whole read, so a *values* dataset of any other class skips it entirely, leaving *depth* and *uncertainty* at their initial null values while line [5] still reports success. The code that renders the grid then walks *depth* over a count it recomputes from the same dimension attributes, so a structurally valid file that declares a non-compound *values* dataset reaches a null dereference with no arithmetic involved. This paper identifies this path from source analysis and does not construct a PoC for it, reporting it as an observation rather than as a measured result. It matters for the design of the fix, because it is closed by the error-propagation change of Section 5.1 rather than by any of the four validation layers, which makes that change an independent requirement instead of a follow-on to the size checks.

As noted before the listing, the implementation follows the specification as written, and none of the three paths is closed by the file format itself. The defect is in the parser’s unchecked arithmetic, and what makes it reachable is that the values driving allocation are taken from the file without validation, rather than any tolerance for malformed encoding.

### 3.5. Proof-of-Concept Construction

A PoC S-102 file was constructed to trigger the integer overflow vulnerability while maintaining a valid HDF5 structure. The construction strategy relies on a distinction between syntactic compliance and semantic inconsistency. The PoC file is syntactically valid, in that it contains properly formatted HDF5 groups, attributes, and datasets that pass standard HDF5 validation tools such as *h5dump* and *h5check*, and its hierarchical structure conforms to the S-102 product specification. However, the file introduces a deliberate mismatch between declared dimension metadata and actual dataset size, creating a semantic inconsistency that the parser does not detect.

This paper built two instances of this trigger that differ only in the embedded *values* dataset. Both select overflow-triggering dimensions, with *numPointsLatitudinal* and *numPointsLongitudinal* set to $2^{30}+4=1073,741,828$ and 4, so that the buffer-size calculation $\mathrm{numLat}\times \mathrm{numLon}\times 2=2^{33}+32$ wraps to 32 in 32-bit arithmetic and the parser allocates only 128 bytes. PoC A, the baseline file *102_real_overflow.h5*, embeds a 1002×1002 compound *values* dataset, about 8 MB of depth and uncertainty data stored in 19,956 bytes after gzip-compressed chunking, so that the subsequent *H5Dread* overflows the 128-byte allocation by the full dataset; this is the file used for the denial-of-service and arbitrary-code-execution campaigns of Section 4. PoC B, the marker file *102_small_spray.h5*, embeds instead a small 32×32 *values* dataset of 8192 bytes filled with the byte 0x41 (ASCII ‘A’), producing the repeated 64-bit value 0x4141414141414141; its 8064-byte overflow of the same 128-byte allocation writes a recognizable pattern into adjacent heap objects, which enables identification of attacker-controlled bytes in CPU registers and crash dumps during the forensic analysis of Section 4.1. Table 3 lists the parameters of both files.

**Figure 2:**
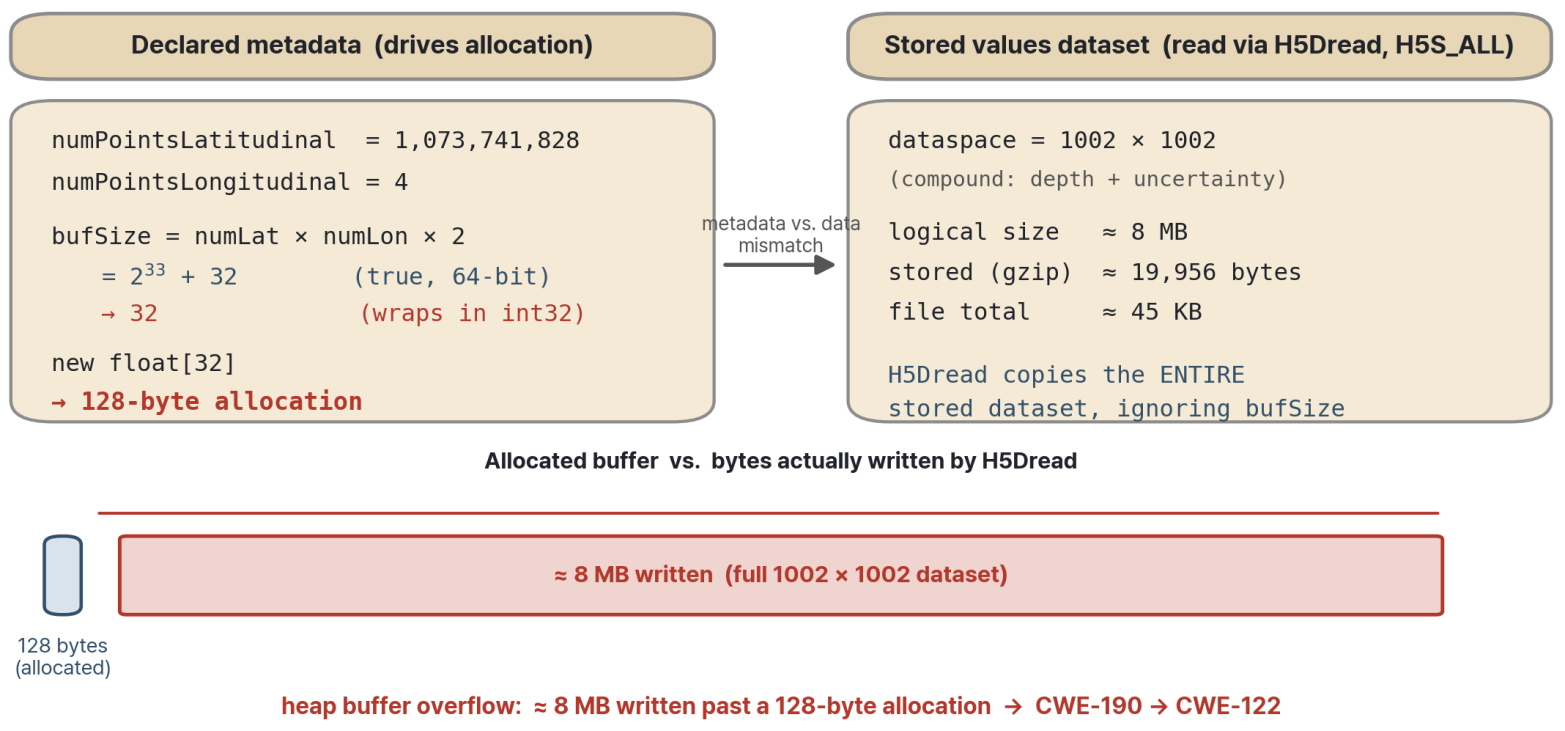
Structure of PoC A, the baseline trigger *102_real_overflow.h5*: the declared metadata drives a large allocation estimate while the stored *values* dataset is much smaller.

In both files, the declared dimensions imply approximately 4.29 billion grid cells, or 8.59 billion depth and uncertainty float values, corresponding to roughly 32 GiB of expected allocation, while the actual *values* dataset is far smaller than that declaration. This inconsistency is invisible to HDF5 structural validation, which checks only that groups, attributes, and datasets are well-formed, not that metadata values are consistent with dataset dimensions. Figure 2 illustrates the structure of PoC A, which totals 45 KB, contrasting the declared metadata that drives parser allocation with the much smaller stored dataset that triggers the buffer overflow.

The base trigger establishes the heap-overflow primitive that produces deterministic DoS on both environments analyzed in Section 4. Escalating it to ACE requires spray engineering calibrated to the heap allocator and ASLR entropy of each environment, and two PoC families were developed for that purpose, one per environment, comprising seven variants for the strong-protection environment. Their construction, spray volumes, and per-variant parameters are withheld under coordinated disclosure, and Section 4 reports only the outcomes.

A second path follows from the same decoupling without relying on arithmetic overflow. Because the original parser sizes its allocation from the declared metadata but invokes *H5Dread* with *H5S_ALL*, a file whose declared dimensions are small, for example 2×2, while its *values* dataspace is larger, for example 32×32, would cause the parser to allocate a small buffer and then read the larger physical dataset, producing the same heap-overflow class. This paper identifies this path from source analysis of the allocation and read logic; it was not separately exercised in the retained experimental artifacts. The Layer 4 cross-check added in Section 5.1 is designed to close it, because *H5Sget_simple_extent_dims* would return dataspace dimensions inconsistent with the declared *numPointsLatitudinal* and *numPointsLongitudinal* before any read. The two paths are therefore complementary: Case 1, the arithmetic-overflow trigger demonstrated above, is rejected by the size-bound and checked-arithmetic guards of Layers 1–3 before any allocation is made; Case 2, the metadata–dataspace mismatch identified from source analysis, is the path the Layer 4 cross-validation is designed to reject.

### 3.6. Experimental Environment

OpenS100 is implemented in C++ using the Microsoft Foundation Classes (MFC) framework and supports multiple S-100 product specifications including S-101, S-102, and S-124. The S-102 parsing module uses HDF5 library version 1.14.6 installed via vcpkg.

To measure the OS-level exploit mitigation effectiveness reported in Section 4, experiments were conducted across two operating system environments hosted on the same physical machine, an AMD Ryzen 7 9800X3D system with 32 GB of memory. Windows 11 Education 24H2, Build 26200, ran as the bare-metal host operating system and provided the strong-protection environment, with default protection mechanisms enabled, including high-entropy heap ASLR on modern Windows, default termination-on-corruption, and the heap walker. Windows 7 Home Basic K x64 SP1 ran as a Hyper-V Type-1 hypervisor guest on the same machine and provided the weak-protection environment, in which heap ASLR provides low entropy and termination-on-corruption is not enabled by default. In this Windows 7 guest, DEP was also disabled at boot (*nx AlwaysOff*); because the successful function-reuse strategy executed no attacker-supplied heap code, this setting was not a precondition for the observed ACE. This configuration is used as a weak-protection reference approximating the heap-ASLR entropy and corruption-policy defaults of pre-Windows-10 systems. It is not a type-approved ECDIS platform and is not presented as a model of any specific deployed installation. It serves to bound the low end of the protection spectrum. Field surveys [22,23,25] document that legacy Windows generations remain present in the fleet, which motivates including a low-protection bound in the experimental design. The dominant strong-vs-weak protection contrast for this vulnerability arises from heap ASLR entropy and corruption-detection aggressiveness, since the pointer-fill PoC chain reported in Section 4.4 achieves code execution through *system()* invocation rather than executing attacker-supplied bytes from the heap. Both environments execute the identical OpenS100 build, MSVC 19.42 x64 Release at commit 2b1dafc1, so ECDIS application behavior is constant across environments. The use of a Hyper-V guest introduces an additional experimental factor that should be distinguished from the guest operating system’s own protection mechanisms. Hyper-V provides each guest with a private virtual address space while mapping guest physical memory onto host-managed physical memory. Consequently, the integer-overflow and heap-buffer-overflow trigger analyzed in this paper remains a guest-process behavior, but virtualization may indirectly affect memory pressure, physical-memory backing, scheduling, and other conditions that can influence allocator state and exploit reproducibility. This paper therefore does not interpret the ACE frequency measured in the Windows 7 guest as an intrinsic or generally applicable exploitation probability for Windows 7 or for legacy ECDIS installations. The virtualized Windows 7 environment is used only as a controlled weak-protection reference, and bare-metal replication would be required to determine whether the measured exploitation frequency changes under a non-virtualized deployment.

The PoC triggers the same integer overflow vulnerability in both environments, but was adjusted per environment for memory layout and library addresses such as *msvcrt!system*. The vulnerability trigger remained identical across environments, but exploit engineering, reachable call sites, and mitigation interactions differed by operating system and exploitation phase. Dynamic analysis was performed using WinDbg and Sysinternals *procdump64* to precisely capture register states, crash locations, and exception codes at the moment of failure. Page Heap was enabled only in supplemental diagnostic runs to confirm heap-corruption behavior. All rates reported in the main experiments were measured under default heap settings without Page Heap.

Reproducibility controls were applied identically across both environments. Each trial used a fresh OpenS100 process launch rather than reusing a crashed process, and no compiler, linker, or kernel mitigation was modified for the reported rates. Default OS protections were preserved throughout. Page Heap was used only in supplemental diagnostic runs to confirm heap-corruption behavior and was not enabled for the quantitative trial counts. Spawn outcomes were detected by parent-chain monitoring for the *cmd.exe* to *calc.exe* sequence, and failure events were captured through *procdump64* together with offline analysis in *cdb* or WinDbg. The weak-protection ACE rate reported in Section 4.4 was obtained from a single fixed-sample campaign of 200 uninterrupted launches within one boot session, with every launch executed and recorded rather than stopping at the first success. Because the launches share one boot session, this paper does not assume that they are mutually independent; the independence question is addressed in Section 4.4.

Section 4 reports the experimental results obtained in this environment. OS protection strength produces two distinct outcomes for the same vulnerability, and neither outcome preserves navigational availability.

## 4. Experimental Results and Exploitability Evaluation

This section reports the empirical evidence for the thesis set out in Section 2.1. The focus shifts from the heap overflow itself, a well-understood vulnerability class, to what happens after the overflow, and to how the system response diverges between OS protection levels.

This paper follows coordinated vulnerability disclosure principles. Detailed exploit artifacts and environment-specific offsets are therefore withheld from the results below to reduce misuse risk, and are available to maintainers under coordinated disclosure. The back matter records the disclosure status.

### 4.1. Exploit Primitive Analysis

To establish that the heap overflow constitutes an exploit primitive rather than a mere crash, this paper measured how attacker-controlled bytes propagate from the corrupted heap buffer into general-purpose CPU registers at the moment of failure. Two complementary measurements were performed, both under the default protection configurations described in Section 3.6, where no compiler-level mitigations were disabled and no kernel-level heap integrity checks were turned off. The first is a marker-propagation measurement using a base-trigger PoC that fills the overflow region with the constant byte 0x41, and the second is a chain-reach measurement using a PoC that fills the overflow region with the structured Pointer Sled pattern described in Section 2.3.3.

The marker measurement uses *102_small_spray.h5*, the marker PoC (PoC B) of Section 3.5, whose *values* dataset is filled with the byte 0x41, the ASCII character ‘A’. Because the overflow rewrites adjacent heap objects with the literal value *0x4141414141414141* interpreted as a 64-bit pointer, any subsequent dereference path that loads from the corrupted region surfaces this pattern in a CPU register. Capturing the crash with *procdump64* and analyzing the dump with *cdb*, this paper observed the marker propagated to multiple registers across multiple PoC dimension variants. Table 4 aggregates the register-level findings, where each row is the register state at the access-violation that ended one trial.

The chain-reach measurement uses the pointer-fill spray PoC against Windows 11 24H2 and was captured with the same *procdump64*/*cdb* workflow. The reference dump captured during one of the 3.3% of baseline trials in which the chain reaches the indirect-call site at *hdf5!H5FD_read+0x4a* shows a register state that corresponds slot-for-slot to the planted block layout. RAX holds the calibrated address of *msvcrt!system* for the boot in question, and the eight bytes at the heap-traced spray slot encode the cycle entries described in Section 2.3.3. The dump confirms that the sprayed layout is reached before the final argument-load sequence. However, immediately before the indirect call, the Windows 11 call site overwrites RCX with a value derived from RBX, which points into the stack. In the captured dump, RCX therefore reflects the post-*mov rcx*, *rbx* stack-derived value rather than a heap address. The captured site executes *call qword ptr [rax+0xB0]* rather than *call rax*: although RAX held the calibrated address of *msvcrt!system*, the processor interpreted the eight bytes at *system+0xB0* as the indirect-call target, and the observed chain failed at this target-resolution step. The preceding *mov rcx*, *rbx* additionally left RCX stack-derived rather than heap-controlled, but the retained dump does not support an interpretation in which *system* was actually invoked, for the reasons Section 4.3 develops under heap–stack isolation. The match between the planted bytes at the spray slot and the call-target register confirms that the spray content drives chain progression rather than being a coincidence of heap layout.

Together, the two measurements establish that the heap overflow provides an exploit primitive rather than a defect-class crash. Attacker-controlled bytes reach pointer-bearing registers as Table 4 shows under default protections, and the spray progresses control flow to a specific indirect-call site whose register state reflects the planted layout. Whether this primitive escalates to ACE depends on the OS-level mitigations analyzed in Section 4.3 and Section 4.4, not on whether mitigations were disabled during measurement.

### 4.2. OS Intervention Mechanism

This subsection establishes how the operating system detects the heap corruption and when in the program flow it terminates the process, distinguishing kernel-initiated process termination from incidental access-violation crashes. All measurements were captured under default Windows 11 24H2 protections using *procdump64* as the dump-monitoring tool and *cdb* from the Windows Software Development Kit (SDK) 10.0.26100 (Debugging Tools for Windows; *cdb* version 10.0.26100.7175) debuggers for offline analysis. No debug-heap mode such as Page Heap was enabled for the rates reported here, and no compiler or linker mitigation was modified.

Across the trials reported in Section 4.3, every captured dump exhibits one of two failure modes that both originate in the post-overflow corrupted heap. The first is a direct access violation, namely *STATUS_ACCESS_VIOLATION* with exception code *0xC0000005*, inside *hdf5.dll* when the parser dereferences a heap pointer that the overflow has overwritten with attacker-controlled bytes, so the faulting address is an attacker-influenced value rather than a random invalid pointer. The second is heap-walker-initiated termination from within *ntdll!RtlReAllocateHeap*, in which the walker detects metadata inconsistency on the next reallocation and the kernel terminates the process directly, bypassing the access-violation path entirely. Neither mode is a generic application bug. Both are the kernel reacting to the post-overflow state of the heap, and both occur deterministically when the vulnerable parser path is exercised.

The second mode is the one that interacts with the dual-outcome trap. In the tested Windows 11 process, termination-on-corruption was active as a kernel heap policy. When heap metadata is determined to be inconsistent with valid heap state, the kernel does not raise an exception that user-mode code could catch, but rather terminates the process directly via *RtlpHeapHandleError*. This termination is non-negotiable at the application level, because no exception handler in OpenS100, the MFC frame, or the parent shell can intercept or recover from it. The output of *!heap -s* on the OpenS100 process before triggering the vulnerability confirms that this policy is active, and this paper did not modify this default in any experiment. This behavior is the Fail-Secure mechanism described in Section 1. The OS detects the post-corruption heap state and prevents ACE by terminating the compromised process, so from the IT security perspective the incident is contained successfully.

### 4.3. Outcome Under Strong Protection, Deterministic Denial of Service

Under Windows 11 24H2, no evaluated exploit variant achieved ACE, and the observed outcomes were dominated by process termination through the failure modes characterized in Section 4.2. The base-trigger PoC *102_real_overflow.h5* crashed in every trial, consistent with a deterministic denial of service. For the function-reuse ACE attempts, a fixed-condition campaign of 180 trials produced no *calc.exe* spawn: 176 trials ended in a crash and 4 neither spawned nor crashed within the monitoring window. All trials were executed on the same default Windows 11 24H2 build with no mitigation flag modified.

The 100% rate against the base trigger reflects the deterministic nature of the vulnerability path, since the code contains no conditional branches between metadata reading and buffer overflow. Once the parser reads the malicious dimension values, the integer overflow occurs unconditionally, the undersized buffer is allocated without validation, and *H5Dread* writes the full dataset regardless of buffer capacity, producing a crash independent of heap layout, memory pressure, or runtime state. ASLR randomized module base addresses between executions as expected, increasing the difficulty of achieving ACE, but had no effect on the DoS outcome because the crash occurs through memory corruption detection rather than through attacker-directed control flow. To verify robustness beyond the primary PoC parameters, testing included multiple dimension combinations that trigger equivalent overflow conditions. Table 5 confirms that the tested dimension pairs triggered the same crash pattern across multiple overflow shapes.

Escalating the same overflow to ACE within this environment did not succeed under the tested strategies. Across the 180 ACE-attempt trials, the absence of any spawn gives a 95% Clopper–Pearson upper bound of 2.03% on the per-trial spawn probability of the tested function-reuse strategy. Three independent mitigation layers cooperate to produce this outcome. The first is high-entropy heap ASLR, which on modern Windows makes the probability that a single planted spray base matches the live heap base very small. The second is the termination-on-corruption heap walker of Section 4.2, which detects the widened corruption that any attempt to raise the matching probability necessarily produces, creating a trade-off between spray spread and chain reach. A quantitative characterization of these two layers across additional operating-system generations is left to future work. The third layer is architectural rather than probabilistic and is analyzed below, because it bears directly on the argument of this paper.

Heap–stack isolation at the indirect-call site is the decisive layer. Chain hijack is reachable at *hdf5!H5FD_read+0x4a* with a measured frequency of 3.3% in the baseline spray configuration, where RAX is loaded with the address of *msvcrt!system*. However, the first-argument register RCX is loaded immediately before the call via *mov rcx*, *rbx*, where RBX holds a stack pointer. As described in Section 4.1, the captured dump shows that by the time the indirect call is reached, RCX has been overwritten by this *mov rcx*, *rbx* reload to a stack-derived value, even though the sprayed layout is still present in heap-traced spray slots. Moreover, the faulting instruction at that site is *call qword ptr [rax+0xB0]*, so the processor dereferences the eight bytes at *system+0xB0*, which lie inside the function body rather than forming a valid code pointer, and the indirect call faults before any transfer to *system* occurs. Because a heap overflow cannot corrupt stack contents, the stack-derived RCX also cannot be made to carry a valid command string. The tested chain therefore does not execute *cmd /c calc*, and no amount of additional heap spraying changes the value loaded from a stack location. This result reflects the evaluated exploitation strategy and does not establish that ACE is impossible under all strategies on Windows 11 24H2; it is empirical evidence that the platform mitigation stack was effective against the evaluated variants.

### 4.4. Outcome Under Weak Protection, Probabilistic Arbitrary Code Execution

Under the legacy Windows 7 SP1 environment, the same integer overflow vulnerability reaches ACE. The path to that result was not direct. Shellcode-oriented attempts, in which a NOP sled precedes injected payload bytes in a sprayed heap region, produced deterministic DoS and zero ACE on both environments. On Windows 11, Data Execution Prevention additionally marks heap pages non-executable. On the Windows 7 guest, where DEP was disabled at boot (Section 3.6), the shellcode strategy nonetheless failed for a structural reason rather than because of DEP: a NOP fill of 0x90 causes the multi-level dereference chain to traverse the invalid literal 0x9090909090909090 and fault mid-chain. The strategy was therefore changed from injecting new code to reusing an already-loaded function, namely *msvcrt!system*, whose invocation spawns a process through the standard cmd /c path without executing any heap byte; because that successful strategy executes no attacker-supplied heap code, the disabled DEP on the Windows 7 guest was not a precondition for it. The technique that stabilizes the dereference chain for this purpose is the Pointer Sled construction of Section 2.3.3, which fills every 8-byte slot of the spray with a pointer back into the spray region so that each intermediate dereference reads a valid heap address; its concrete parameters are withheld under coordinated disclosure.

**Figure 3:**
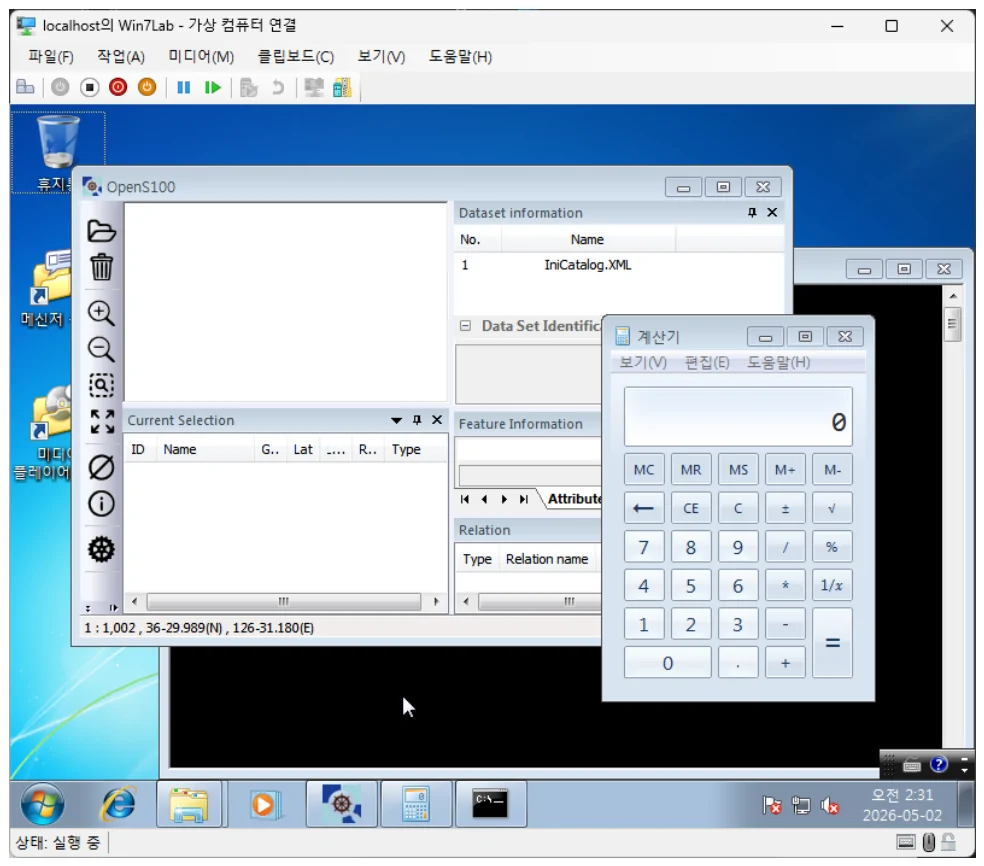
Arbitrary code execution on the Windows 7 SP1 weak-protection reference environment.

The vulnerability trigger is identical to the strong-protection environment described in Section 3.4, where integer overflow in *numLat* × *numLon* × *sizeof(struct)* produces an undersized buffer allocation. The write that overflows that buffer is issued by HDF5 while materializing the dataset described in Section 3.4, and on chunked datasets this materialization proceeds through chunk decompression, which is the write observed at the corruption site. The overflow corrupts a *file_ptr*-adjacent structure such that offset 8 of that structure contains an attacker-controlled pointer to the spray region. In the Windows 7 SP1 environment, this corruption surfaces at an indirect-call site inside *hdf5.dll*, from which a multi-level pointer dereference dispatches the calibrated address of the C runtime command-string dispatcher. Successful trials were confirmed by out-of-process parent-chain monitoring, which verified that the parent of the spawned *calc.exe* was the just-spawned *cmd.exe* rather than *explorer.exe* or *services.exe*, distinguishing the spawn from any pre-existing or user-initiated calculator instance. Concrete offsets and address values are withheld pending coordinated disclosure. A representative successful spawn is shown in Figure 3: loading a crafted PoC S-102 file in OpenS100 triggers the heap overflow and the *Pointer Sled* chain, which reuses *msvcrt!system* to spawn the Windows Calculator (*calc.exe*) via cmd /c. In that screenshot the *OpenS100* window’s status bar shows the 1002-point grid of PoC A, and the surrounding window chrome is the Korean-language Hyper-V guest console of the test virtual machine (top menu, left to right: File, Action, Media, Clipboard, View, Help), which is part of the test harness rather than OpenS100.

In a fixed-sample campaign of 200 uninterrupted launches, with every launch executed and recorded, 14 ACE events were observed, an empirical per-launch rate of 7.0% with a 95% Clopper–Pearson confidence interval of [3.9%,11.5%]. This campaign replaces an earlier aggregation whose denominator reflected planned rather than executed launch counts. The 200 launches used a single exploit configuration, the Pointer Sled of Section 2.3.3, with a fresh process launch per trial inside one Windows 7 SP1 boot session, and the campaign ran to completion rather than stopping at the first success. Of the 200 launches, 14 produced a confirmed *calc.exe* spawn, 181 crashed, and 5 neither spawned nor crashed within the monitoring window, so a memory-corruption outcome occurred in 195 launches (97.5%). Fixing a single exploit configuration, rather than pooling the exploratory and working variants used in the earlier aggregation, is what makes the per-launch rate a well-defined binomial estimate. Under an illustrative Bernoulli model that assumes independent launches and a stable per-launch probability equal to the observed 0.07, the cumulative probabilities are those in Table 6; these are model-based illustrations rather than experimentally measured operational exploitation probabilities.

The 200 launches were conducted within a single boot session, so this paper treats 0.07 as an empirical per-launch rate specific to that session and configuration rather than as a session-independent probability. Allocator and ASLR state may retain session-level structure, and this paper does not claim that launches across reboots are identically distributed or mutually independent. Heap ASLR in Windows 7 SP1 provides low entropy, so a planted spray base matches the live heap base with a non-negligible per-launch probability, consistent with the observed rate. A malicious S-102 file persisted in the dataset directory is auto-loaded on each startup, so a repeatedly rebooted deployment would present repeated opportunities; whether successive reboots behave as independent trials is not established here and is identified as future work in Section 6.4. Under the illustrative independent-trial model above, such repetition would accumulate a low per-launch probability into a higher cumulative probability over an operational lifetime, which suggests that OS-level defenses optimized for always-on IT environments may be misaligned with the repeatedly booted nature of OT environments. The interpretation of these outcomes for maritime safety and availability requirements, including the dual-outcome trap framing and the Common Vulnerability Scoring System (CVSS) severity envelope, is taken up in Section 6.

### 4.5. Comparison with Prior Work

Table 7 compares this vulnerability with the Lua interpreter vulnerability reported in prior work [1]. Read together, the two show that both active and passive content can affect S-100 implementations.

## 5. Mitigation Evaluation and Recommendations

The dual-outcome trap discussed in Section 6.1, supported by the empirical results of Section 4, shows why the operating system cannot resolve the conflict. It acts only once memory has already been corrupted. Under strong protection, OS termination prevents ACE but causes a loss of availability, while under weak protection, OS detection is bypassed and ACE is achieved. Placing the check in the application layer, where input validation rejects the offending values before allocation, removes both outcomes without depending on post-corruption OS intervention.

### 5.1. Application-Level Defense-in-Depth

A patched version of OpenS100 was developed implementing defense-in-depth controls. The primary patch modifies *S102_VG_BathymetryCoverage.cpp*, specifically the *Read()* function where the vulnerable size calculation occurs. The patch implements a four-layer defense-in-depth strategy, shown in Listing 2.

**Listing 2.** Illustrative four-layer validation strategy.// Patched S102_VG_BathymetryCoverage.cpp #include <cstddef> #include <vector> #if defined(_MSC_VER) #include <intsafe.h> #endif // Portable checked size_t multiply (true on overflow) static bool ck_mul(size_t a, size_t b, size_t∗ r) { #if defined(__GNUC__) || defined(__clang__)     return __builtin_mul_overflow(a, b, r); #elif defined(_MSC_VER)     return FAILED(SizeTMult(a, b, r)); #else     if (a && b > SIZE_MAX/a) return true;     ∗r = a ∗ b; return false; #endif } // Layer 1: reject invalid dimensions if (numLat <= 0 || numLon <= 0) {     H5Tclose(type); H5Dclose(dsID); return false; } size_t lat = (size_t)numLat, lon = (size_t)numLon; // Layer 2: checked arithmetic for BOTH products size_t nPoints, byteSize; if (ck_mul(lat, lon, &nPoints)) {     H5Tclose(type); H5Dclose(dsID); return false; } // Layer 3: resource bound (reject before allocating) constexpr size_t MAX_GRID_POINTS = 100’000’000; if (nPoints > MAX_GRID_POINTS) {     H5Tclose(type); H5Dclose(dsID); return false; } // Element size from the file’s compound type; reject if // invalid, and check the byte-size product as well size_t elemSize = H5Tget_size(type); if (elemSize == 0 || ck_mul(nPoints, elemSize, &byteSize)) {     H5Tclose(type); H5Dclose(dsID); return false; } // Type-safe allocation: raw bytes, no float-layout assumption std::vector<std::byte> buf(byteSize);

Listing 2 is written to be internally safe rather than merely indicative. It applies checked arithmetic to both the point-count and the byte-size products, rejects an invalid element size, and allocates a raw byte buffer so that no relationship between the HDF5 element size and *sizeof(float)* is assumed. The Layer 4 cross-check in Listing 3 likewise checks every HDF5 return value and verifies the dataspace rank before comparing dimensions. Appendix A collects additional engineering practices beyond this minimal validated pattern, such as wrapping the HDF5 handles in RAII types so that the close calls shown here cannot be missed on future code paths.

Layer 2 employs checked arithmetic to detect integer overflow at the point of computation, independent of the target architecture’s word size, using *__builtin_mul_overflow* on GCC and Clang and *SizeTMult* from the *intsafe.h* SDK header on MSVC. Unlike the original code’s implicit reliance on *size_t* width, which prevents overflow only on 64-bit platforms but silently wraps on 32-bit *size_t* environments, checked arithmetic rejects overflowing products on any architecture. For the demonstrated PoC, the product is computed exactly in *size_t* and rejected by the maximum-grid-size bound at Layer 3, while for larger inputs that would overflow *size_t* the checked multiplication rejects the calculation directly.

**Listing 3.** Illustrative HDF5 metadata cross-check (Layer 4).// Layer 4: HDF5 dataspace cross-check (required) hid_t spaceID = H5Dget_space(dsID); if (spaceID < 0) {     H5Tclose(type); H5Dclose(dsID); return false; } // Reject unless the dataspace is 2-D, then read extents if (H5Sget_simple_extent_ndims(spaceID) != 2) {     H5Sclose(spaceID); H5Tclose(type); H5Dclose(dsID);     return false; } hsize_t dims[2]; if (H5Sget_simple_extent_dims(spaceID, dims, NULL) < 0) {     H5Sclose(spaceID); H5Tclose(type); H5Dclose(dsID);     return false; } // Declared metadata must match the physical dataspace if (dims[0] != (hsize_t)numLat ||     dims[1] != (hsize_t)numLon) {     H5Sclose(spaceID); H5Tclose(type); H5Dclose(dsID);     return false;  // Metadata mismatch - reject } H5Sclose(spaceID);

The buffer size calculation in the patched code replaces the hardcoded * 2 multiplier with the HDF5 type introspection Application Programming Interface (API) *H5Tget_size(type)*, which returns the exact byte width of the compound data type as stored in the file. This removes the dependency on a fixed two-float element layout and keeps the calculation correct even when the S-102 compound type definition changes in future editions of the product specification.

The 100-million-point limit in Layer 3 derives from S-102 Product Specification guidance. Section 11.2.2 recommends approximately 600×600 grid cells, or 360,000 points, per coverage, corresponding to an uncompressed file size of approximately 10 MB. The patch threshold of $10^{8}$ points provides a safety margin exceeding 270 times the recommended value. Legitimate high-resolution datasets are therefore accepted without false rejection, while resource exhaustion attacks, which require dimension products on the order of $2^{30}$ or above, are rejected.

The fourth layer is required to close the metadata–dataspace mismatch path, namely Case 2 of Section 3.4, and is included in the recommended patch. It performs library-level cross-validation by verifying that declared metadata matches the actual HDF5 data structure, as shown in Listing 3.

This cross-check addresses the discrepancy between how the vulnerable code determines buffer size versus how *H5Dread* determines read size. The original code uses *H5S_ALL* for both dataspace parameters, meaning HDF5 reads the entire dataset as defined by the file’s dataspace structure, not by any application-level metadata. An alternative approach replaces the *H5S_ALL* pattern entirely by creating explicit dataspaces matching validated dimensions, so that *H5Dread* reads exactly the expected number of elements regardless of actual dataset size. Production deployments should consider implementing both mechanisms, since cross-validation detects suspicious files for security audit, while range limitation keeps operation safe even when detection logic contains errors.

A secondary patch in *S102_FI_BathymetryCoverage.cpp* restores proper error propagation. In the original code, the call site discards the return value of the grid read and returns *true* unconditionally, so a read that populated nothing is indistinguishable from one that succeeded, and the consuming code reaches a null pointer. The patch captures that return value and propagates it to the caller. This closes the third path noted in Section 3.4 as well as the rejection paths opened by the four validation layers, which is why it is required rather than optional.

### 5.2. Patch Verification

Verification testing was conducted using the Windows SDK debugger *cdb.exe* with the original PoC file across both environments, in three configurations. The unpatched original raised a second-chance *C++ EH exception e06d7363* and crashed from heap corruption, an intermediate build carrying only the 64-bit arithmetic fix raised an *access violation c0000005* and crashed from a null pointer, and the full four-layer patch reported a load failure and continued running.

The intermediate result revealed a secondary issue, in that without the error propagation patch, the application attempted to access uninitialized depth arrays, causing a null pointer crash. The full patch chain ensures errors propagate correctly through the call stack. Table 8 extends this verification to the broader input categories used to evaluate the patched parser, including the Case 2 metadata–dataspace mismatch path identified through source analysis and a benign S-102 sample exercised for functional regression.

**Figure 4:**
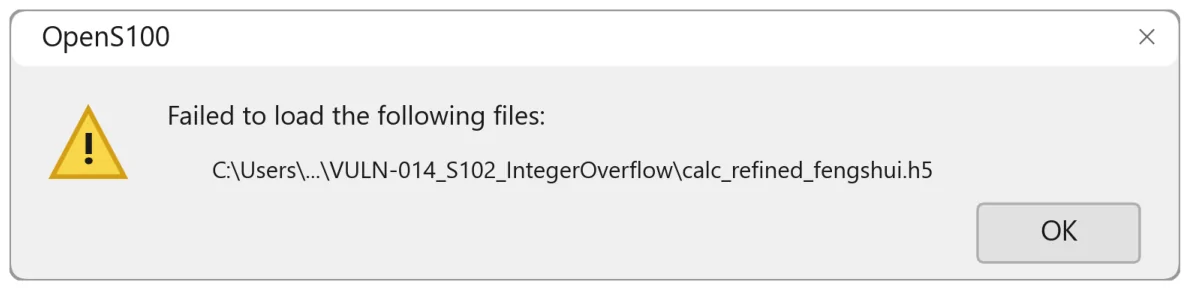
Patched OpenS100 rejecting the malicious S-102 file.

Figure 4 shows the patched application rejecting the malicious S-102 file while maintaining normal operation.

The patch eliminates both attack outcomes of the dual-outcome trap simultaneously. Table 9 summarizes the security impact across both environments.

The patch verification demonstrates that application-level input validation prevents both branches of the dual-outcome trap. The OS heap integrity check is not triggered because no corruption occurs, eliminating the strong-protection DoS branch, and no exploitable primitive is created, eliminating the weak-protection ACE branch. The patched application gracefully rejects malicious files and continues operating, satisfying both the IT security objective of preventing memory corruption and the OT safety objective of maintaining continued availability, resolving the dual-outcome trap by moving defense from the OS layer to the application layer where the trap does not exist.

The patch adds constant-time arithmetic checks, HDF5 metadata queries, and error-path propagation before the dominant O(n) dataset read, so its runtime cost is expected to be low relative to the subsequent dataset parsing workload. To quantify this expectation on representative S-102 workloads, this paper measured wall-clock parse time on three valid grids on the same desktop host described in Section 3.6, comparing an unpatched parse path that trusts metadata against a patched path that executes the four validation layers before allocation. The purpose of this measurement is not to establish performance equivalence between the two paths, but to characterize the computational cost that validation adds; it should be read as a focused microbenchmark rather than as an end-to-end benchmark of a production ECDIS. The microbenchmark was implemented in Python 3.13.7 (h5py 3.15.1 built against HDF5 1.14.6, NumPy 2.1.3) using h5py to isolate the HDF5 dataset-read workload rather than to reproduce full OpenS100 execution. The cost was measured at the H5Dread workload level because the added checks are scalar dimension validation, checked arithmetic, an upper-bound comparison, and a single HDF5 dataspace metadata query that do not iterate over grid cells, whereas the dominant cost is the dataset read that both paths execute identically. The added checks are therefore expected to remain approximately constant with respect to grid cell count, while the dataset-read cost grows with the amount and storage layout of grid data. Each workload was benchmarked under 100 interleaved A/B repeats on the validated grid after a five-iteration warm-up, with timing values reported as medians and standard deviations of the per-repeat wall-clock parse time, together with the mean paired (patched minus unpatched) difference and its 95% confidence interval. Table 10 reports the result.

Pairing the patched and unpatched runs by repeat yields mean overheads of −0.003, +0.007, and −0.028 ms across the three workloads, with 95% confidence intervals of [−0.019,+0.012], [−0.006,+0.021], and [−0.177,+0.122] ms, respectively, computed as Student’s *t* intervals on the paired per-repeat differences; a non-parametric bootstrap agrees to within 0.001 ms. Every interval spans zero and is at most about 0.3 ms wide, bounding any mean validation cost well below the per-repeat timing variability. This paper does not interpret the near-zero or negative point estimates as speedups, nor an interval that contains zero as proof of statistical equivalence; the intervals indicate only that the incremental validation cost did not dominate total parse time in the tested workloads, consistent with validation that adds metadata-level checks rather than a second pass over the grid. These workloads are examples consistent with the constant-cost structure described above; they do not establish identical performance across all grid sizes, shapes, or storage configurations, and broader benchmarking on additional S-102 geometries would further characterize end-to-end parser performance. Measurements on representative embedded or type-approved ECDIS hardware were not feasible in this paper, because such systems are proprietary and were not available for experimental modification or instrumentation; validation on operational ECDIS hardware therefore remains future work requiring cooperation with equipment manufacturers or system integrators.

### 5.3. Fail-Soft Parser Isolation for OEM Deployments

Application-level validation is the first line of defense because it prevents corruption before the OS must choose between termination and non-detection. Where not already implemented, OEM ECDIS architectures can further reduce the availability impact of residual parser defects that escape validation. S-100 product parsing can be isolated into a low-privilege helper process separated from the primary ECDIS human–machine interface through a broker/target model. If the helper process fails while parsing an S-102 product, the ECDIS should disable only the affected bathymetry overlay, preserve the last-known-good navigation display, log the rejected product, and alert the operator. This architecture complements parser validation, since validation prevents the demonstrated vulnerability, while process isolation and graceful degradation reduce the operational consequence of future parser defects. In the terminology of Section 2.1, such isolation provides Fail-Soft behavior at the application level, which contributes to, but does not by itself constitute, a Fail-Safe state at the navigation-system level.

### 5.4. Implications for Implementation Guidance

As the S-100 family matures, parser-validation expectations can be stated alongside the data models the product specifications already define. The draft clauses below are illustrative wording, offered to the relevant working groups as one way such guidance might read in S-102 and S-100 Part 15.

1. **Dimension validation.** An S-102 parser shall validate *numPointsLatitudinal* and *numPointsLongitudinal* before using them in allocation, indexing, loop bounds, or HDF5 read operations.
2. **Checked arithmetic.** An S-102 parser shall perform checked arithmetic for all element-count and byte-size calculations derived from file metadata. Overflow or truncation shall cause graceful rejection of the input.
3. **Metadata–dataspace consistency.** Before invoking *H5Dread* with *H5S_ALL* or an equivalent full-dataset read, an S-102 parser shall verify that declared grid dimensions are consistent with the HDF5 dataspace dimensions returned by the HDF5 API.
4. **Resource limits.** Product specifications shall define maximum accepted grid dimensions or implementation-defined upper bounds sufficient to prevent memory-exhaustion attacks.
5. **Graceful rejection.** Validation failure shall result in logged rejection of the affected product or layer, not process termination of the ECDIS application.

Section 4 showed this empirically. The results suggest value in expressing this validation expectation independently of platform-level protections, whether through implementation guidance, product specifications, or type-approval practice. OS-level Fail-Secure responses through process termination conflict with maritime requirements for continued availability, and on legacy deployments still active in the fleet, those protections are weaker still.

IEC 61174 type approval can incorporate malformed-input testing such as fuzzing and boundary condition testing. This paper does not identify a publicly documented IEC 61174 requirement for malformed-input resilience testing; if such provisions exist in current or draft revisions, the items below should be read as complementary to them. Specifically, type approval can verify the following.

- Testing with malformed S-100 data products containing boundary and overflow values.
- Verification that parsers reject malicious inputs without crashing, under both modern and legacy OS configurations.
- Validation that security failures result in graceful degradation rather than process termination.
- Demonstration that defense does not depend on OS-level mitigations alone.

Because openly available implementations are read and reused, publishing the validated parsing template alongside OpenS100 [33] would let the safe pattern travel through the same channel that carries the current one. The four-layer defense-in-depth patch presented in this paper provides a concrete model for secure S-102 parsing that other implementers can adopt. The patch is independent of compiler-, linker-, and OS-level mitigations, so protection is uniform regardless of the deployment environment, a property that the dual-outcome trap analysis in Section 4 establishes as essential for maritime CPS.

## 6. Discussion and Limitations

This section interprets the empirical results of Section 4 through the dual-outcome-trap and CVSS lenses, then addresses methodological constraints and outlines future research directions for maritime software security.

### 6.1. The Dual-Outcome Trap

The central interpretive finding of this paper is that a Fail-Secure response at one layer does not by itself establish a Fail-Safe state at the system level. The same OS-initiated process termination is a process-level security success and, at the same time, a loss of the navigation service whose system-level safety consequence is not settled by the termination alone. ACE achieved where OS protection is weak is a second, distinct failure. The two outcomes occur under opposite protection regimes, and neither, on its own, yields a Fail-Safe navigation state.

From the IT security perspective, the heap integrity check in the strong-protection environment detected memory corruption and terminated the process before the attacker could redirect execution flow. ACE was prevented, so a process-level Fail-Secure response was achieved. In the weak-protection environment, the check failed and ACE was achieved, which from the IT perspective is a failure of defense.

From the maritime CPS perspective, termination of the ECDIS process is a safety-relevant loss of navigation functionality rather than merely an application-availability event, and this interpretation does not require assuming that every ECDIS failure immediately produces an accident. IMO ECDIS performance standards require independent backup arrangements so that safe navigation is not compromised in the event of ECDIS failure, including timely transfer to the backup system during critical navigation situations [9], and IMO guidance treats ECDIS software and hardware malfunctions as conditions that may present a risk to maritime safety [35]. Loss of ECDIS functionality is therefore a failure mode that maritime standards explicitly require systems to tolerate through backup and continuity arrangements. During pilotage in restricted waters, that transition is time-critical, since the navigator must recognize the failure, transfer to backup, and re-establish situational awareness while the vessel continues to move. In OpenS100, and in ECDIS architectures that auto-load the configured product directory at startup, the installed chart database is loaded by enumerating every S-100 data product in that directory and parsing each file. If a malicious S-102 file resides in this directory, the parser encounters the crafted metadata during the auto-loading sequence and crashes before the navigator gains access to any interactive interface. A further consequence follows, derived from the documented auto-loading architecture rather than observed on a vessel installation. A watchkeeper or an automatic watchdog restart would re-trigger the identical crash, and the system stays inoperable until the malicious file is removed. In the weak-protection environment, the outcome, in which the attacker executes arbitrary commands within the ECDIS process, creates a different but equally severe violation in which the navigator continues to operate a compromised system without indication, while display falsification, position-data tampering, and data exfiltration all become possible.

Three levels should be kept distinct. The experiment reproduced termination of the OpenS100 process and the associated loss of navigation-display functionality; it did not reproduce a collision, grounding, or ship-level loss of safe navigation aboard an operational vessel. The safety relevance of that functionality loss is established normatively, by the IMO backup and malfunction-handling requirements cited above, rather than by an accident reproduced here. The operational consequences on a specific vessel, such as added workload or temporary loss of situational awareness during restricted-water navigation, depend on the backup architecture, bridge procedures, and circumstances of the transit, and were not experimentally measured in this paper.

From the attacker’s perspective, the deterministic DoS outcome requires no shellcode, ROP chain, heap spray, or ASLR bypass, since crafted dimension metadata alone is sufficient to trigger process termination. For the Windows 7 SP1 ACE outcome, the attack does not require injected shellcode or ROP, but it does require function-reuse-oriented spray construction, calibrated call-target addresses, and probabilistic alignment against the legacy ASLR layout. The attacker manipulated metadata values in an S-102 file that is structurally valid at the HDF5 level; under the pre-signing or compromised-signing scenarios of the threat model, equivalent content could also carry a valid Part 15 signature. In the strong-protection environment, the OS detects the resulting heap corruption and terminates the process. In the weak-protection environment, the OS fails to detect the corruption and the attacker obtains direct execution. Either outcome compromises the maritime mission.

The paradox is one of location rather than strength. A defense that acts only after memory has been corrupted has no response left that preserves both safety and availability, and no adjustment of detection sensitivity changes that.

Table 11 evaluates each defense layer through the lens of this paradox. The OS-layer mechanisms interact asymmetrically, since LFH and ASLR raise the complexity of ACE but do not prevent DoS, while heap integrity checking terminated the process in the tested campaigns, suppressing the evaluated ACE attempts. Only application-level validation prevents both ACE and DoS before corruption occurs. Conversely, the layers where defense could prevent both ACE and DoS without process termination, namely the application layer and the HDF5 library layer, provide no protection in the current implementation.

### 6.2. CVSS Assessment

Severity was assessed using CVSS version 3.1 [36]. The dual-outcome trap analyzed in this paper produces two distinct attack scenarios that warrant separate scoring, namely deterministic DoS on Windows 11 24H2 and probabilistic ACE on Windows 7 SP1. Each scenario is further evaluated under two delivery models. The first is local file delivery, with Attack Vector Local, representing USB or already-present malicious data, and the second is network delivery via SECOM automatic update channels, with Attack Vector Network, representing supply-chain compromise of an authorized distribution tier. Table 12 summarizes the four resulting scenarios. In the vector strings, AV denotes Attack Vector, AC Attack Complexity, PR Privileges Required, UI User Interaction, S Scope, and C, I, and A the Confidentiality, Integrity, and Availability impacts, respectively.

A 7.0% per-launch spawn rate would compound across repeated ECDIS reboots under the illustrative independent-trial model of Section 4.4, in which roughly 50 launches yield about 97% probability of at least one spawn, provided launches are independent and the per-launch probability remains stable; inter-reboot independence is not established, so this is a model-based illustration rather than a measured operational probability. This compounding behavior is an operational/temporal property of the deployment, not a property that the CVSS Attack Complexity metric captures, and this paper therefore presents it as an operational threat-model note rather than as a CVSS metric value. For example, this paper does not advance AC from H to L on cumulative grounds. The corresponding cumulative-trials severity should be interpreted alongside the CVSS Base scores rather than as a substitute for them.

Three properties of these scores bear on their interpretation. First, the same primitive yields a Medium score under one OS and a High score under another, which the CVSS framework cannot natively express in a single number, so the vulnerability is most accurately represented as a *score envelope* parameterized by deployment context rather than as a fixed value. Second, the per-trial Base score for the Win7 ACE scenario retains AC:H because Attack Complexity is defined as a property of a single exploitation attempt, and the cumulative-trials behavior stays in the operational note above rather than being propagated into the metric value. Third, the framework does not capture the amplified real-world impact in maritime CPS contexts where a “Medium” availability score translates to potential loss of primary navigation capability during pilotage in restricted waters, and a CPS-specific extension incorporating delivery trust, exploitation persistence, and physical safety consequence is deferred to future work, as Section 6.4 discusses. Accordingly, the disclosure report will provide multiple vector candidates so that the CNA and downstream Computer Security Incident Response Team (CSIRT) triage can select the score appropriate to each deployment context.

### 6.3. Broader Implications

Before discussing broader implications, this paper delimits what was and was not demonstrated. The experimentally demonstrated findings are limited to the OpenS100 S-102 implementation and the tested software and operating-system configurations; no commercial ECDIS product was evaluated, and this paper does not establish that any commercial implementation contains the same defect. The broader relevance is structural rather than implementation-specific: any parser that derives allocation sizes from untrusted metadata without checked arithmetic, bounds validation, and metadata-to-dataspace consistency checks may be exposed to the same vulnerability class, and other gridded S-100 products may present comparable validation requirements where they use related metadata-driven allocation. These are parser-hardening implications for the wider S-100 ecosystem, not experimentally demonstrated vulnerabilities in untested products.

The findings of this paper raise several broader implications that extend beyond the specific S-102 integer overflow vulnerability. When combined with the Lua scripting vulnerability demonstrated in prior work [1], the results show that both active and passive content can create security-relevant attack surfaces during processing, so parser validation remains relevant even for data products that contain no executable code.

The underlying issue concerns the relationship between data authentication and processing safety. The S-100 Part 15 digital signature scheme was designed to ensure that data products originate from authorized hydrographic offices and remain unmodified during transmission. Deployment practice may treat authenticated data as safe to process, but S-100 authentication guarantees provenance rather than parser safety. Treating authenticated data as safe to parse is a reasonable reading of the standard as written rather than a deployment error. This distinction lies outside the scope that the standard explicitly addresses. This paper’s findings show what follows from conflating the two, in that a structurally valid HDF5-encoded S-102 input with inconsistent metadata can trigger memory corruption in a parser that does not independently validate that relationship before allocation; under the delivery scenarios of Section 3.2, such a file could also carry a valid signature without that signature attesting to its parser safety. The authentication layer and the parsing safety layer address orthogonal concerns, and signature verification does not provide guarantees about parser memory safety. This distinction is not unique to maritime systems. Similar assumptions have been challenged in other domains where signed software updates or authenticated data feeds were weaponized, as the SolarWinds [28,32] and ASUS Live Update [29] supply chain compromises demonstrate. The findings highlight the distinction between authenticating a product’s origin and establishing that it can be parsed without adverse effects, and the latter can be addressed through parser-level validation.

The allocation pattern identified in S-102 is not confined to a single parser function. Source code analysis of OpenS100 at commit *2b1dafc1* reveals that S-102 is currently the only HDF5 grid parser fully implemented, as shown in Table 13. S-104 and S-111 use related HDF5 grid-coverage structures and dimension metadata patterns, so a future implementation that derives allocation sizes from such metadata without checked arithmetic or metadata-to-dataspace validation could be exposed to the same vulnerability class. This is a structural inference from the product data model and allocation pattern; no S-104 or S-111 parser vulnerability was experimentally demonstrated in this paper. As implementations expand to additional gridded S-100 products ahead of the IMO 2029 milestone, consistent parser-validation practices matter more. Section 5.1 sets out one such practice in full.

The dual-outcome trap documented in Section 4 reflects a deeper structural problem in how security technologies are adopted in safety-critical CPS. Operating system security mechanisms such as heap integrity checking, ASLR, and DEP were developed for enterprise IT environments where the primary objectives are preventing unauthorized access and code execution. In these environments, terminating a compromised process is a proportionate and effective response. However, when these same mechanisms are inherited by OT systems through the use of general-purpose operating systems, their Fail-Secure behavior creates an exploitable structure in which the attacker need not defeat the security mechanism but can deliberately trigger it, knowing that the security response itself will disrupt the safety-critical operation. Conversely, when these mechanisms are absent or weakened, as in legacy OS deployments still common in the maritime fleet, the same vulnerability reaches ACE through probabilistic exploitation, producing a different but equally severe form of safety violation. This structural difference is also reflected in threat classification frameworks, since the same attack is classified as T1499 Endpoint DoS under ATT&CK Enterprise but as T0829 Loss of View under ATT&CK for ICS [30]. This classification difference illustrates how an availability event can translate into a physical safety impact in CPS contexts.

Attack complexity and defense complexity are asymmetric. The attack requires only metadata-level manipulation using common HDF5 tooling, and the resulting file passes the structural HDF5 checks evaluated in this paper, while valid S-100 authentication is treated separately as a threat-model condition. A comprehensive defense, by contrast, may span parser implementation, testing practice, deployment configuration, and operational handling.

The dual-environment design highlights a mismatch between OS mitigation assumptions and the operational pattern of repeatedly restarted CPS deployments. ASLR was designed to defeat single-shot exploitation attempts in always-on enterprise systems. In repeatedly restarted safety-critical CPS such as ECDIS, however, each fresh process launch provides a new opportunity for heap-layout variation, though the degree of statistical independence between launches was not measured. Under an explicit independent-trial assumption, which this paper does not claim to have established across reboots, repeated launches can convert a low per-launch probability into a high cumulative probability over an operational lifetime. This observation suggests that mitigation design assumptions, formed in the IT context, require re-examination when transferred to OT contexts, where temporal patterns differ in kind, not merely in degree.

Taken together, these observations point past the specific defect examined here. A substantial and growing number of S-100 product specifications are under development, and those built on gridded coverages share the grid metadata structure that drives allocation in S-102. The results therefore motivate a validation procedure that can be reused across gridded product parsers, together with tooling that automates it, rather than treating the present patch as specific to one function. The four-layer validation sequence developed in Section 5.1 is the core logic such a tool would implement, and its portability across products follows from the shared grid metadata structure it validates against. A Data Bill of Materials, extending Software Bill of Materials principles to navigation data products by declaring expected dimension bounds and dataset sizes that parsers verify before processing, would supply that tooling with the per-product expectations it needs, and would place the declaration at the point in the distribution chain where the producer still knows what the correct values are.

### 6.4. Limitations and Future Work

This paper analyzed a single reference implementation, OpenS100 at commit *2b1dafc1* dated 29 April 2026, rather than multiple commercial ECDIS systems. This constraint arises from the proprietary nature of commercial ECDIS software, resource limitations, and regulatory restrictions. The findings are therefore statements about this codebase: the behavior was found and reproduced in OpenS100, and nothing here characterizes any commercial ECDIS product. What generalizes is not the defect but the validation principle, which applies to any parser that sizes an allocation from metadata the file supplies. OpenS100 is openly available and is listed as an interim S-100 infrastructure component in IHO document HSSC16-04.5A [37], so the pattern it embodies is visible to other implementers; whether any particular implementation carries hardening of its own is unverified here, and multi-implementation confirmation is deferred to future research.

Exploitation testing spanned two OS environments, and the rates reported in Section 4 are specific to the hardware, build, and execution configurations described in Section 3.6. In particular, the Windows 7 SP1 weak-protection environment was executed as a Hyper-V guest rather than on bare metal. Although the vulnerability trigger and the guest-visible memory-corruption path occur within the guest process, virtualization can alter physical-memory backing, resource pressure, scheduling, and potentially allocator-dependent execution conditions. This paper therefore treats the measured ACE frequency as an empirical result of this specific virtualized configuration, not as a general exploitation probability for Windows 7 or deployed ECDIS systems. Replication on bare-metal legacy platforms would be required to quantify any virtualization effect. Generalizability to other HDF5 versions, compiler toolchains, OS builds, hypervisors, or bare-metal configurations has not been independently verified, and the precise probability under intermediate configurations such as Windows 10 LTSC and Server 2016 remains unmeasured.

The boot-crash loop scenario described in Section 6.1 is grounded in the documented ECDIS auto-loading architecture and the observed parser behavior, but was not directly reproduced on a physical vessel installation. The scenario is a plausible consequence of the vulnerability given the architectural design of modern ECDIS startup sequences, yet empirical confirmation on a production system would strengthen the safety impact claim. Similarly, the assertion that S-104 and S-111 parsers would inherit the same vulnerability class rests on source code analysis of the shared HDF5 grid coverage structure rather than on tested parser implementations, as those parsers exist only as feature catalogue stubs in the current OpenS100 codebase. The patch overhead reported in Table 10 was measured on a desktop host on three representative valid grids and was below the per-repeat measurement noise. End-to-end benchmarking on embedded ECDIS hardware, including cold-disk and integrated OpenS100 chart-load measurements, was not feasible here because type-approved ECDIS platforms are proprietary and were not available for experimental modification or instrumentation, and it remains future work requiring cooperation with equipment manufacturers or system integrators.

Vulnerability discovery relied on expert manual code review rather than systematic coverage-guided fuzzing with tools such as AFLNet [20]. This approach identified the integer overflow vulnerability, but the issues found through manual review should be regarded as a lower bound. A standalone fuzzing harness isolating S-102 parsing functions from the MFC application, potentially using mutation strategies that systematically vary HDF5 metadata fields including boundary-condition dimension values, would enable comprehensive dynamic analysis and is recommended as future work.

This paper did not evaluate non-Windows platforms, alternative allocators, or non-MSVC toolchains. The integer-overflow-to-undersized-allocation pattern is language- and parser-level, but exploitability and post-corruption outcomes are platform-specific. On platforms with different allocator behavior, different HDF5 builds, or different mitigation stacks, the same parser defect may manifest as a crash, safe allocation failure, or a different exploit primitive. The proposed validation patch is platform-independent because it rejects malicious metadata before allocation and before any OS-specific mitigation is invoked.

Two directions follow most directly from these limitations. Establishing how widely the metadata-driven allocation pattern appears would require examining S-102 parsers beyond the one analyzed here, which depends on collaboration with implementers and on access arrangements for proprietary code, in the spirit of the industry cooperation that IACS Unified Requirements E26 and E27 introduced in 2022 [6,7].

Separately, the CVSS framework does not capture the amplified impact of a “Medium” availability score in safety-critical CPS contexts, where loss of primary navigation translates directly to physical risk. A complementary scoring system incorporating delivery trust, exploitation persistence, and physical safety consequence would be valuable, and would need formal specification, pilot deployment, and evaluation for possible use in maritime standards and assurance frameworks.

## 7. Conclusions

This paper has demonstrated that passive S-102 data products containing no executable code can compromise the memory safety of the tested OpenS100 S-102 implementation. An end-to-end S-100 Part 15 signing-and-verification experiment was not performed; the results instead show why authenticity and parser safety should be treated as separate properties, since signature verification establishes provenance without evaluating whether the authenticated payload can be parsed safely. A PoC file with manipulated grid dimension metadata triggered an integer overflow in the parser’s buffer size calculation, producing a heap buffer overflow without any executable payload. The immediate cause is an unchecked integer multiplication in the OpenS100 S-102 parser, reachable because S-102 Section 10.2.1 mandates the metadata attributes that drive memory allocation while bounds validation, checked arithmetic, and metadata-to-dataspace cross-validation are matters the current specification leaves to the implementation, consistent with the scope Part 15 sets for itself. The specification does not prescribe the pre-allocation validation procedure absent from the analyzed implementation, and any parser that treats dimension metadata as trustworthy without adding validation of its own can exhibit the same condition. Where prior work asked whether S-100 content can be executed unsafely [1], the question here is how such content can be parsed resiliently.

The central result concerns the dual-outcome trap that emerges once the overflow is triggered, and both of its branches conflict with the maritime requirement for continued availability. Under Windows 11 24H2 with strong memory protection, no tested function-reuse variant produced code execution and the corrupted OpenS100 process terminated in almost all trials, so the OS satisfied the IT Fail-Secure objective while eliminating the primary navigation display during pilotage. Under Windows 7 SP1 with weak protection, the same overflow reached ACE probabilistically once the strategy shifted from injected shellcode to existing-function reuse, leaving the navigator operating a compromised system without indication. The asymmetry in attacker effort is worth noting, since the deterministic DoS outcome requires no shellcode, ROP chain, heap spray, or ASLR bypass, whereas the ACE outcome requires calibrated call-target addresses and probabilistic alignment against the legacy layout. The dual safety violation arises not from the strength of OS defense but from its location, and no adjustment of detection sensitivity can resolve that.

Because operating-system mechanisms act only after corruption, the check has to sit earlier. The four-layer patch of Section 5 adds constant-time arithmetic checks, HDF5 metadata queries, and error-path propagation before the dominant O(n) dataset read, and it removes both branches at once: no corruption occurs, so the OS heap integrity check is never reached, and no exploitable primitive is created, because the malicious metadata is rejected before allocation. Verification across both environments confirmed that the patched application rejects malicious files and continues operating, and on the three grids of Table 10 the measured overhead was below the per-repeat measurement noise, with end-to-end benchmarking on embedded ECDIS hardware deferred as future work. S-100 is still maturing, and these results provide empirical evidence that may inform implementation guidance as the family grows. The experimentally demonstrated findings of this paper are limited to the OpenS100 S-102 implementation evaluated here; their relevance to other gridded S-100 products follows from shared metadata-driven parsing structures and should be understood as a design-level inference rather than evidence of a demonstrated vulnerability in those products. Future revisions of Part 15 and of individual product specifications may consider specifying application-level input validation, safe integer arithmetic, metadata-to-dataspace cross-validation, and graceful error handling independently of any operating system protections, and IEC 61174 type approval can likewise incorporate parser testing under both modern and legacy OS configurations.

## Figures and Tables

**Table 2:** Vulnerability trigger conditions.

Condition	Requirement
File Type	S-102 Bathymetric Surface product only
File Structure	Must contain BathymetryCoverage HDF5 group
Metadata	numPointsLatitudinal, numPointsLongitudinal attributes present
Overflow Trigger	Product of numLat, numLon, and 2 exceeds 32-bit integer range
Example Values	numLat is 4 and numLon is 1,073,741,828

**Table 3:** Parameters of the two overflow-trigger Proof-of-Concept files. PoC A is the baseline trigger used in the exploitation campaigns; PoC B is the marker file used for register-level forensics. Both declare the same overflow-inducing dimensions and therefore produce the same 128-byte undersized allocation.

Parameter	PoC A (Baseline)	PoC B (Marker)
File	102_real_overflow.h5	102_small_spray.h5
numPointsLatitudinal	1,073,741,828	1,073,741,828
numPointsLongitudinal	4	4
Buffer-size product (×2)	$2^{33}+32\to 32$	$2^{33}+32\to 32$
Undersized allocation	128 bytes	128 bytes
Actual *values* dataspace	1002×1002	32×32
Dataset, logical	∼8 MB	8192 bytes
Dataset, stored	19,956 bytes (gzip)	8192 bytes
Overflow volume	∼8 MB	8064 bytes
0x41 marker	none	0x4141414141414141
Role	DoS/ACE campaigns	register forensics

**Table 4:** Register state at the access violation that ended each trial.

Register	Observed Value	Implication
rax	0x4141414141414141	Read-target pointer attacker-controlled
rcx	0x4141414141414141	Pointer base attacker-controlled
rbx	0x4141414141414141	Object pointer attacker-controlled
r8	0x4141414141414141	Write value attacker-controlled
r9	0x4141414141414141	Write-target pointer attacker-controlled

**Table 5:** Denial-of-Service reproducibility across four dimension variants.

Dimensions	Buffer	Overflow	Result
$4\times (2^{30}+4)$	128 B	8 KB	Crash
65,537×65,537	1 MB	7 MB	Crash
$2\times (2^{30}+8)$	128 B	8 KB	Crash
$8\times (2^{29}+2)$	128 B	8 KB	Crash

**Table 6:** Illustrative cumulative probability under an independent-trial model, using the observed session-specific per-launch rate of 0.07.

Trial Count *N*	P(≥1 Spawn)
10	51.6%
20	76.6%
30	88.7%
50	97.3%

**Table 7:** Comparison with the prior Lua scripting vulnerability [1].

Dimension	Lua (Prior)	S-102 (This Paper)
Attack surface	Active (scripts)	Passive (data)
Vulnerability class	Design flaw	Memory corruption
Root cause	Missing sandbox	Implementation flaw enabled by S-102 parser-safety gap
Strong-protection result	ACE achieved	No ACE in 180 tested exploit attempts; base trigger produced deterministic DoS
Weak-protection result	—	ACE 7.0% (Pointer Sled)
RCE (network)	Yes	Potential
DoS achieved	Yes	Yes; 100% for base-trigger trials
CVSS score	9.3	5.5–8.1 (envelope, Section 6.2)

**Table 8:** Patch test matrix for benign and malicious S-102 inputs.

Input (Purpose)	Unpatched Behavior	Patched Behavior
Benign S-102 sample (functional regression)	Loaded normally	Loaded normally
Case 1 overflow PoC (integer-overflow path)	Heap corruption/crash	Rejected before allocation
Case 2 mismatch (metadata–dataspace, source analysis)	Heap-overflow class (not separately exercised)	Addressed by HDF5 cross-validation (Layer 4)
Malformed HDF5 file (API error path)	Undefined or crash-prone path	Rejected with error propagation
Oversized but non-overflowing grid (resource exhaustion)	Excessive allocation attempt	Rejected by maximum grid bound

**Table 9:** Patch effect on both experimental environments.

Metric	Pre-Patch Behavior	Patched (Both Environments)
numLat × numLon	Wraps to 16 in 32-bit arithmetic	4,294,967,312, computed exactly in size_t
Bounds check	None	Rejected because size > MAX_GRID_POINTS
Integer overflow	Occurs on both environments	Prevented by checked arithmetic
Heap buffer overflow	Occurs on both environments	Prevented
Allocation behavior	Undersized 128-byte allocation, 8 KB write → heap overflow on both environments	Rejected by overflow/size validation
Naive 64-bit widening	On Win11, possible ∼32 GiB allocation attempt or null-pointer crash on *new[]* failure	N/A (rejected before allocation)
OS-detected DoS	100% process termination on Win11	None (no corruption to detect)
Probabilistic ACE	7.0% per launch on Win7 SP1	None (no exploit primitive)
Application stability	Crash on Win11, and crash or compromise on Win7 SP1	Stable, malicious file rejected

**Table 10:** Parse time with and without validation on the tested desktop workloads, reported with the mean paired (patched − unpatched) difference and its 95% confidence interval.

Workload	File Size	Unpatched (ms)	Patched (ms)	Mean Diff. [95% CI] (ms)
600 × 600 contiguous	2.88 MB	0.795 ± 0.069	0.792 ± 0.066	−0.003 [−0.019, +0.012]
600 × 600 chunked	2.89 MB	1.577 ± 0.097	1.578 ± 0.103	+0.007 [−0.006, +0.021]
2000 × 2000 chunked	32.03 MB	11.885 ± 0.467	11.855 ± 0.644	−0.028 [−0.177, +0.122]

**Table 11:** Defense layer effectiveness against the dual-outcome trap.

Layer	Blocks ACE	Blocks DoS	Net Effect
Application validation	Yes (if implemented)	Yes (if implemented)	Both prevented
Library (HDF5)	No	No	Neither
Compiler/GS	N/A (heap vuln)	No	Neither
Linker (ASLR)	Partial (raises bar)	No	DoS only
Linker (DEP)	Partial	No	DoS only
LFH/heap layout randomization	Raises ACE complexity	No	DoS remains
Heap integrity/termination-on-corruption	Yes, in tested runs	No	No ACE in tested runs; process killed
DoS prevention	None	None	No layer addresses DoS

**Table 12:** CVSS 3.1 vectors and base scores for the four delivery scenarios.

Scenario	Vector String	Base Score	Severity
Win11 DoS, local delivery	AV:L/AC:L/PR:N/UI:R/S:U/C:N/I:N/A:H	5.5	Medium
Win11 DoS, SECOM auto-load	AV:N/AC:L/PR:N/UI:N/S:U/C:N/I:N/A:H	7.5	High
Win7 ACE, local, single trial	AV:L/AC:H/PR:N/UI:R/S:U/C:H/I:H/A:H	7.0	High
Win7 ACE, SECOM auto-load, single trial	AV:N/AC:H/PR:N/UI:N/S:U/C:H/I:H/A:H	8.1	High

**Table 13:** HDF5-based S-100 parser implementation status in OpenS100.

Product	Data Type	Parser Status	Vulnerability
S-102	Bathymetric Surface	Implemented	Patched
S-104	Water Level	FC only	N/A (not yet implemented)
S-111	Surface Current	FC only	N/A (not yet implemented)

## Data Availability

Data are available on request due to privacy, legal, and ethical restrictions. The OpenS100 source code is publicly available at https://github.com/S-100ExpertTeam/OpenS100, accessed on 2 May 2026, and the analyzed commit is dated 29 April 2026. The vulnerability was reported to the OpenS100 maintainers before submission. At the time of writing, CVE assignment and upstream patch integration are pending. Detailed exploit artifacts and environment-specific offsets are withheld to reduce misuse risk and are available to maintainers under coordinated disclosure.

## Abbreviations

The following abbreviations are used in this manuscript:

ACE	Arbitrary Code Execution
API	Application Programming Interface
ASLR	Address Space Layout Randomization
CPS	Cyber–Physical System
CVSS	Common Vulnerability Scoring System
CWE	Common Weakness Enumeration
DEP	Data Execution Prevention
DoS	Denial of Service
ECDIS	Electronic Chart Display and Information Systems
ENC	Electronic Navigational Chart
HDF5	Hierarchical Data Format version 5
IACS	International Association of Classification Societies
IEC	International Electrotechnical Commission
IHO	International Hydrographic Organization
IMO	International Maritime Organization
IT	Information Technology
KHOA	Korea Hydrographic and Oceanographic Agency
LFH	Low Fragmentation Heap
MFC	Microsoft Foundation Classes
OEM	Original Equipment Manufacturer
OT	Operational Technology
PoC	Proof-of-Concept
RCE	Remote Code Execution
RENC	Regional ENC Coordinating Centre
ROP	Return-Oriented Programming
SDK	Software Development Kit
SECOM	Secure Communication Between Ship and Shore
SOLAS	Safety of Life at Sea
USB	Universal Serial Bus
VAR	Value Added Reseller

## Appendix A. Hardening Items for HDF5-Based S-100 Grid Parsers

The four validation layers of Section 5.1 address the vulnerability class analyzed in this paper. The items below are general hardening practices for parsers that read HDF5-based S-100 grid products, independent of any particular implementation, and are listed so that the compact listing in Section 5.1 is not mistaken for a complete production template.

- Wrap HDF5 handles in RAII types so that *H5Tclose*, *H5Dclose*, and *H5Sclose* cannot be skipped on error paths.
- Check the return value of every HDF5 API call rather than only the calls in the validation sequence.
- Query *H5Sget_simple_extent_ndims* before assuming a two-dimensional dataspace.
- Apply checked multiplication to both *numLat* × *numLon* and the subsequent *size* × *elemSize*.
- Avoid the *bufSize/sizeof(float)* cast pattern where the compound-type element size is not guaranteed to be a multiple of *sizeof(float)*.
